# A comprehensive investigation of geoenvironmental pollution and health effects from municipal solid waste landfills

**DOI:** 10.1007/s10653-024-01852-4

**Published:** 2024-02-23

**Authors:** Anna Podlasek, Magdalena Daria Vaverková, Aleksandra Jakimiuk, Eugeniusz Koda

**Affiliations:** 1 Department of Revitalization and Architecture, Institute of Civil Engineering, Warsaw University of Life Sciences – SGGW, Nowoursynowska 159, 02 776 Warsaw, Poland; 2 Department of Applied and Landscape Ecology, Faculty of AgriSciences, Mendel University in Brno, Zemědělská 1, 613 00 Brno, Czech Republic

**Keywords:** Waste management, Pollution, Heavy metals, Carcinogenic effects, Spatial distribution, PCA analysis

## Abstract

This study investigates human health risks associated with heavy metals (HMs) occurrence in municipal solid waste (MSW) landfills. For testing of selected MSW landfills steps were involved, including site characterization, soil sampling and chemical testing, statistical analysis, as well as health risk assessment, carcinogenic and non-carcinogenic effects. For the Polish landfill (Radiowo) the average HMs concentrations were found in the following order: Zn (52.74 mg/kg DM) > Pb (28.32 mg/kg DM) > Cu (12.14 mg/kg DM) > Ni (4.50 mg/kg DM) > Cd (3.49 mg/kg DM), while for the Czech landfill (Zdounky): Zn (32.05 mg/kg DM) > Cu (14.73 mg/kg DM) > Ni (4.73 mg/kg DM) > Pb (0.10 mg/kg DM) = Cd (0.10 mg/kg DM). Strong positive correlations between selected HMs demonstrated identical origins. Principal component analysis (PCA) performed for the Radiowo landfill transferred the soil parameters into three principal components (PCs), accounting for 87.12% of the total variance. The results of the PCA analysis for the Zdounky landfill revealed three PCs responsible for 95.16% of the total variance. The exposure pathways of HMs for landfills were in the following order: ingestion > dermal absorption > inhalation. For both landfills, the values of hazard quotient were lower than 1, indicating no potential negative health effects. In terms of the hazard index (HI), for both landfills, no adverse human health effects occur (HI < 1). The incremental lifetime cancer risk (ILCR) values indicated negligible or acceptable carcinogenic risk of HMs (average ILCR in the range from 5.01E−10 to 5.19E−06).

**Supplementary Information:**

The online version contains supplementary material available at 10.1007/s10653-024-01852-4.

## Introduction

Municipal solid waste (MSW) landfills play a significant role in waste management systems (He et al., 2022; Matheson, 2022; Vaverková, 2019, 2023). Nevertheless, landfills, even well-designed and secured, can pose potential risks to both the environment and human health, particularly because of the presence of heavy metals (HMs) in the waste stream (Aendo et al., 2022; El Fadili et al., 2022; Koda et al., 2020a; Podlasek et al., 2021; Siddiqua et al., 2022). Typical sources of HMs in landfills may be incinerator ashes, mine wastes and household materials (i.e., electronic equipment, batteries, paints, inks, leather, rubber, photographic films, and steel products) (Choudhury et al., 2022; Jakimiuk et al., 2022). Debnárová and Weissmannová (2010) pointed out that Cd may originate in batteries, coatings of plastics and pigments, as well as automotive radiators, electronics, tires, gasoline, and oils. The occurrence of HMs in soils adjacent to landfills may also be the result of leachate formation and subsequent migration from the landfill body (Yeilagi et al., 2021). Major factors influencing contaminants release and increase in HMs concentrations in soil are related to waste disposal, leachate and geothermal wastewater discharge, the energy sector, and the automotive industry (Kosowski et al., 2019; Trach, 2020; Tucki et al., 2020; Wang et al., 2020). Zhou et al. (2022) highlighted that the primary source of HMs could be associated with the siting and operation of waste disposal facilities such as waste treatment centres, domestic waste incineration plants, and leachate treatment stations.

The hazards associated with HMs occurrence are the effects of their persistence (Ali et al., 2019), toxicity, and vulnerability to accumulation (Liu et al., 2020). The persistence allows HMs to remain in the ecosystem for an extended period, thereby increasing the likelihood of exposure and potential adverse effects (Emenike et al., 2021). This means that landfill sites can harm surrounding soils, water resources, and human health, and can persist even after landfill closure and reclamation (Koda, 2012; Morita et al., 2021). Makuleke and Ngole-Jeme (2020) indicated that even more than 20 years after the landfill closure, the leachate migration around the landfill may be in progress. Nevertheless, the intensity of this migration decreases over time, and as evidenced by Barlaz et al. (2002), a decrease of 75% may be observed in the first year, and even 90% during the 4 years after closure. Iravanian and Ravari (2020) found that in the post-closure phase, several years after the closure of the landfill, elevated concentrations of HMs (Fe, Zn, Ni, Cu) in soils are still measured.

Considering that landfills may coexist with agricultural areas, the risk of their negative impact on these areas should not be overlooked (Vaverková, 2019, 2023). As reported by Kicińska and Wikar (2021), increased concentrations of Cr and Ni may be measured in agricultural soils owing to the use of phosphate fertilizers and municipal wastewater. It is therefore crucial to be aware of the adverse effects of landfills on food safety and sustainable agriculture (Pysarenko et al., 2022).

Furthermore, HMs exhibit toxicity, which means that, even at low concentrations, they can have harmful effects. These effects can range from damaging cellular structures and impairing physiological functions to disrupting vital biochemical processes within organisms (Priya et al., 2022). HMs can bind to soil particles, sediments, or other matrices, leading to their gradual build-up over time (Ore & Adeola, 2021). The magnitude of HMs accumulation (along with that of other contaminants) is influenced by soil physicochemical parameters (Fronczyk et al., 2016; Sieczka & Koda, 2016; Nartowska et al., 2017). Consequently, the vulnerability to accumulation increases the potential for prolonged exposure and associated health risks (Nkwunonwo et al., 2020; Obiri-Nyarko et al., 2021).

Given the above, it is justified to research human health risk assessment of HMs pollution to evaluate the magnitude and extent of contamination and determine the potential impacts on both the environment and human well-being (Vaverková, 2019, 2023). Human exposure to HMs from contaminated soils is a significant concern, particularly for individuals residing in proximity to MSW landfills (Ihedioha et al., 2017). These exposure pathways include direct contact with contaminated soil, inhalation of particles, consumption of contaminated food crops, and bioaccumulation of HMs in the human body (Ali et al., 2021), and therefore, they should be thoroughly investigated. Several researchers have focused on the visible pollution of HMs in operational and non-operational landfill areas. Therefore, the design of barrier systems is recognized as an important approach to mitigate their negative impact (Hussein et al., 2021; Jakimiuk, 2022; Koda, 2012).

The main objective of this study was to investigate the hazards associated with the occurrence of HMs in the vicinity of MSW landfills. The study acknowledged that, although the tested landfills are classified in the same group, they can differ in terms of their current exploitation phase (operational or non-operational), management rules, applied protection systems, and composition of waste stored. We hypothesize that these differences may have implications for the presence and potential risks of HMs. The specific objectives of the study were: (1) to assess HMs pollution in reference to environmental regulations and using pollution indices, (2) to identify the spatial distribution and possible sources of HMs in soils at different MSW landfill sites, (iii) to evaluate carcinogenic and non-carcinogenic risks of HMs exposures through soils ingestion, inhalation, and dermal absorption.

By conducting an in-depth human health risk assessment, this study provides valuable insights into the extent of HMs contamination and its implications for waste disposal sites. This knowledge builds the foundation for implementing appropriate mitigation strategies, such as remediation measures, waste management improvements, and regulatory actions, to safeguard both the environment and public health.

## Material and methods

### Description of the study areas

#### Radiowo landfill

**Fig. 1:**
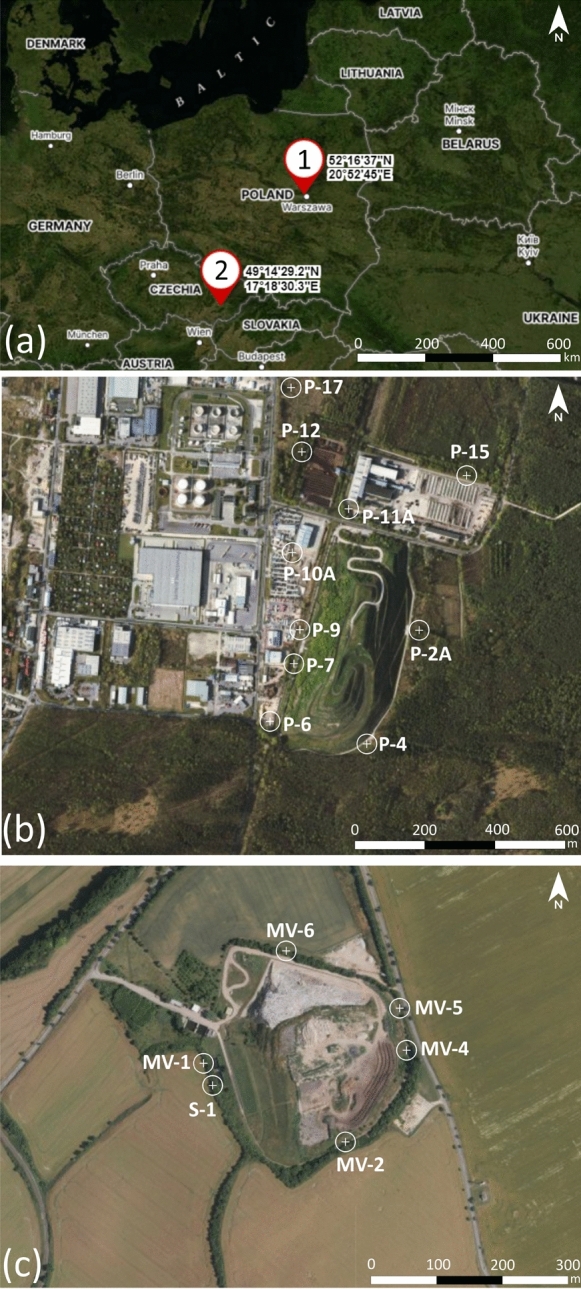
Study areas on the background of surrounding countries (**a**); location of the Radiowo landfill (**b**); location of the Zdounky landfill (**c**)

The Radiowo landfill (52° 16′ 37″ N, 20° 52′ 45″ E) is located in the municipality of Stare Babice (in the Klaudyn village area) and partially in the city of Warsaw (Bemowo District), Poland (Fig. 1b).

The landfill is surrounded by forests of the “Bemowo” Forest Park from the south and east (including the “Kalinowa Łąka” floristic nature reserve and the “Łosiowe Błota” peatland reserve). On the northern side, the landfill borders the green-waste composting plant (until 2012 DANO system). Near the landfill, there is a railway siding on the western side, beyond which there are industrial facilities. At a distance of approximately 200 m from the foot of the slope, there is a paved area used as a customs car depot, and in the northwest direction, there is a fuel storage warehouse. Approximately 350 m north of the green-waste composting facility (around 400 m from the landfill), the Lipkowska Woda Stream flows, which is a tributary of the Zaborowski Canal. At a distance of approximately 550 m from the composting facility, there is the closest residential area. A larger housing estate is located approximately 1.5 km eastwards from the site. Kampinos National Park is located approximately 3 km northwest of the landfill.

The Radiowo landfill covers an area of approximately 16 ha and has a height of 60 m. Until 1991, the Radiowo landfill stored unsorted MSW from the Warsaw districts over approximately 30 years. Since 1992, the facility has been exclusively used as a landfill to incorporate screening residues from the Radiowo Composting Plant (DANO system). Only waste materials (screening residues) from compost production are deposited, which serves as a shaping material for the landfill. These include plastics, films, tires, textiles, scrap metal, and a small amount of organic waste (approximately 5%).

The types and morphologies of waste destined for disposal at the Radiowo landfill are presented in the Supplementary materials (Table S1). Some parts of the waste stream have been utilized in the technological processes (Table S2) associated with shaping the landfill body, constructing covering and reclamation layers, stabilizing slopes, and building drainage and leachate collection systems (Koda et al., 2020b).

At the time of its establishment, the landfill had not been lined at the base. The first aquifer is found at a depth of 0.5–2.0 m below the surface level (b.s.l.) and was exposed to contamination from leachate for many years. The second aquifer is located at a depth of 15–25 m b.s.l. and is isolated from the surface by layers of glacial till, and locally by clay deposits. A detailed description of the hydrogeological conditions of the Radiowo area can be found in previous studies (Koda, 2012; Koda et al., 2016; Podlasek et al., 2021).

In 1997–1998, construction projects for landfill reclamation were developed. Leachate collection systems were installed at the base of the landfill slopes, and excess rainwater was collected from the composting facility. These waters were managed on the landfill within a closed-loop system (recirculation). A vertical barrier was constructed as part of the remediation, which reached the impermeable layer to prevent leachate migration. The leachate collection system was also implemented, consisting of sealed tanks, retention ditches, finger drains, cascade tanks on the landfill's crest, and a micro-irrigation system on selected slopes. In November 2001, the leachate management was extended to include pre-treated rainwater from the composting facility, which had previously been discharged into the Zaborowski Canal. The landfill body was shaped considering stability conditions and the final land use plan, including the implementation of biological covers on the slopes and further recreational applications. Additionally, a landfill gas management system was installed, and adjacent drainage ditches were restored. In 2016, waste disposal at the Radiowo landfill ceased, indicating that the landfill stopped accepting any further waste deposits. Subsequently, in 2017, the landfill was officially closed, signifying the end of its operation as a waste disposal site. It is planned to develop the landfill for recreational purposes, mainly for skiing in winter and mountain biking in summer.

#### Zdounky landfill

The Zdounky landfill (49° 14′ 29.2″ N 17° 18′ 30.3″ E) is located in the area of Nětčice, part of the Zdounky municipality, in the Kroměříž District (Zlín Region) in the Czech Republic (CR). It was established on approximately 10 ha of agricultural land. Currently, landfills occupy an area of approximately 7 ha, and each side is surrounded by farmlands (Fig. 1c). During its construction, the Zdounky landfill was designed to handle a waste volume of 907,000 cubic meters, to serve a population of 75,000 individuals. However, the landfill has received a total amount of 1,280,750 Mg of waste to date. The landfill is categorized as a sanitary landfill. It is designed to store S-category waste, specifically sub-category S-OO3. This classification refers to “other waste”, which includes a range of materials, that is, biodegradable organic substances. The landfill is equipped to handle and store various types of waste in a controlled and environmentally responsible manner. Hazardous wastes are not deposited in the Zdounky landfill. The facility focuses on accepting nonhazardous waste materials (Table S3). Additionally, biogas generated from the decomposition of waste within the landfill is collected and processed in a motor-generator unit. This process converts biogas into electrical energy, which can be utilised for various purposes. Moreover, a part of the landfill body crown is operated as a composting plant. This means that organic waste, such as food scraps or garden waste, may be processed through composting methods. This helps to manage and reduce organic waste and, promotes environmentally friendly practices within the landfill site. On a designated part of the Zdounky landfill site, there is a recycling area in which inert demolition waste material is processed and stored. This recycling area serves the purpose of segregating and managing specific types of waste, particularly inert materials from demolition activities. The Zdounky landfill is designed as an engineering facility that incorporates a sealing system to minimize the potential for leachate leakage into the surrounding environment (Podlasek et al., 2023). This sealing system consists of multiple layers, including a mineral liner composed of practically impermeable soil, with a thickness of 1 m; a high-density polyethylene (HDPE) geomembrane located on the top of a mineral liner, with a thickness of 1.5 mm; and a drainage layer above the geomembrane, composed of sands and end-of-life tries, which helps to properly manage the leachate generated in the landfill body. The reclamation of the Zdounky landfill took place between 2017 and 2019. The planned service life of the landfill is extended, and it is expected to continue operating until 2027. Owing to its location within agricultural areas, the Zdounky landfill may pose a potential risk of soil–water contamination in the surrounding areas. This risk may be of particular importance as food crops may accumulate high amounts of HMs from contaminated soils and leachate.

### Soil sampling and analysis

Soil samples were collected and stored, transported and prepared for laboratory analysis following the procedures described in PN-ISO 10381–1. (2008), PN-ISO 10381–2 (2007), PN-ISO 10381–3 (2007), PN-ISO 10381–5 (2009). For both landfills, samples were collected from the top layer (0–0.25 m) of the selected soil profiles. The sampling points were strategically distributed based on their proximity to the piezometers, which monitor the groundwater quality at the landfill sites (Fig. 1b, c). The grain size distribution was analyzed according to PN-EN ISO 14688–1 (2018). Before HMs content analysis, soil samples were digested using a Milestone microwave oven (Start D, Italy), following the procedure described in Method 3051A (USEPA, 2007). The digested soil-acid solutions were filtered and adjusted to 100 mL with deionized water. The concentrations of HMs (Ni, Cd, Pb, Zn, and Cu) were measured using Atomic Absorption Spectrometry (AAS) in an air-acetylene flame. HMs analysis was performed in an iCE 3000 spectrometer (Thermo Scientific, USA). Thermo SOLAAR software allowed quick and easy optimization of the method. A hollow cathode lamp with each metal was used at the specific wavelength. For the analysis of each of the HMs, the Calibration Curve Method was applied (Farrukh, 2012). The method involved the preparation of standard solutions with five different concentrations. The absorbance of these standard solutions was measured using a spectrophotometer at a specific wavelength, and the recorded absorbance values were used to create a calibration curve. A regression line was fitted to the data points to establish the relationship between concentration and absorbance (Draghici et al., 2011). Once the calibration curve was prepared, a test solution was adjusted to fall within the measurable range of the calibration curve. The adjusted test solution's absorbance was then measured at the same wavelength as the standard solutions. The concentration of the HM in the test solution was determined by referencing the calibration curve. The actual HM content in each sample was calculated based on the results of the spectrometry study (Adegboye et al., 2021):

1 $$Content\;of\;HM \left( {\frac{{{\text{mg}}}}{{{\text{kg}}}}} \right) = \frac{{Concentration\;of\;HM\;in\;solution\; \left( {\frac{{{\text{mg}}}}{{\text{L}}}} \right) \times volume \;of\;dilution\; \left( {\text{L}} \right) \times 1000}}{{weight\; of\;sample \left( {\text{g}} \right) }}$$

The analysis was performed in triplicates for each soil sample tested. All the chemicals used were of analytical reagent grade. Soil pH was analyzed using the method described by PN-EN ISO 10390. (2022). The interpretation of soil quality based on pH was performed according to the classification presented by Bruce and Rayment (1982). The electrical conductivity (EC) was measured using the conductometric method. For both the pH and EC analyses, a multimeter CX-601 (Elmetron, Poland) was used. Salinity levels were assessed based on EC measurements in soil–water extractions. The interpretations of the EC values were in accordance with Richards (1954).

### Pollution assessment

For the Polish landfill site, the soil quality was assessed using HMs content based on permissible values reported in the Regulation of the Minister of the Environment (Journal of Laws No., 2016 item 1395) (Table S4).

For the Czech landfill site, the HMs concentrations were compared with those of Decree No. 153/2016 Coll. on the establishment of details concerning the quality of agricultural land, issued by the Ministry of the Environment of the CR. Moreover, the target and intervention values of HMs concentrations were considered in reference to the legal guidelines of selected countries (Table S5).

For further comparative analysis, the literature data on HMs content in soil were also included, considering the average abundance of total HMs in typical soils, background levels of HMs in soils, and relative contents of HMs in soils (Hazelton & Murphy, 2016).

### Statistical analysis

The results of the soil quality analysis were subjected to statistical processing using the Statistica 12 software (StatSoft Inc., Tulsa, OK, USA). Descriptive statistics, including mean, median, minimum, maximum, standard deviation, variance coefficient of variation (CV), skewness, and kurtosis, were calculated. The normality of the datasets was checked using the Shapiro–Wilk test. The Mann–Whitney U test and Student t-test were applied to check the significance of the differences between the analyzed soil features of the Radiowo and Zdounky landfills. The Mann–Whitney U test was used in the case of lack of normality of datasets, while the Student t-test was used when normality existed. Levene and Brown-Forsythe tests were used to determine the equality of variances. The interpretation of the results was based on the guidelines reported by Rabiej (2018).

Pearson’s correlation coefficients (r) were calculated to determine the relationships between the soil parameters (Schober et al., 2018).

Principal Component Analysis (PCA) was performed to explain the variance of interrelated soil features (Subba Rao et al., 2020) and to reduce the dimensionality of the soil monitoring datasets for both landfills. This tool has also been used to identify possible sources of HMs in soils (Obiri-Nyarko et al., 2021). Varimax rotation was performed using Kaiser’s criterion with an eigenvalue greater than 1.0 (Kaiser, 1958). Before PCA analysis, the soil data were standardized. According to Maji and Chaudhary (2019), the variables were found as statistically significant when the correlation coefficient was higher or equal to 0.5.

### Health risk indices

To assess non-carcinogenic and carcinogenic risks, a computational approach of USEPA (1989) was adopted, including the source of pollution, exposure routes, and receptors. The following routes were considered: (1) soil ingestion, (2) inhalation of soil particles, and (3) dermal absorption. The average daily dose (ADD) from each exposure route was determined using the formulas:

#### Direct soil ingestion (ADD_ing-soil_)

2 $$ADD_{ing - soil} = \left( {\frac{{C_{soil} \times IngR_{soil} \times EF \times ED \times CF}}{BW \times AT}} \right)$$

where C_soil_—concentration of HM in soil (mg/kg DM), IngR_soil_—soil ingestion rate (mg/day), EF—exposure frequency (day/year), ED—exposure duration (year), CF—conversion factor (kg/mg), BW—average body weight (kg), AT—average time (day).

#### Inhalation of soil particles (ADD_inh_)

3 $$ADD_{inh} = \left( {\frac{{C_{soil} \times InhR \times EF \times ED}}{BW \times AT \times PEF}} \right)$$

where InhR—inhalation rate (m^3^/day), PEF—particle emission factor (m^3^/kg).

#### Dermal absorption (ADD_der_)

4 $$ADD_{der} = \left( {\frac{{C_{soil} \times AF_{soil} \times SA \times ABS \times EF \times ED \times CF}}{BW \times AT}} \right)$$

where AF_soil_—skin adherence factor (mg/cm^2^/day), SA—exposed skin area (cm^2^), ABS—dermal absorption factor (–).

#### Hazard quotient (HQ)

To estimate the potential non-cancerogenic health risk of the soil HMs the formula for calculation Hazard Quotient (HQ) was used (USEPA, 1989):

5 $$HQ = \frac{ADD}{{RfD}}$$

where RfD—reference dose (mg/kg/day).

#### Hazard index (HI)

The overall potential risk posed by HMs was calculated using the formula of Hazard Index (HI), including the sum of HQ for each HM analyzed (USEPA, 1989):

6 $$HI = \mathop \sum \limits_{i = 1}^{n} HQ$$

For the interpretation of *HQ* and *HI* values for each route of exposure, it was considered that no adverse human health effect occurs when *HQ* ≤ 1 or *HI* ≤ 1, and there is a potential non-carcinogenic risk when *HQ* ≥ 1 or *HI* ≥ 1 (USEPA, 1989).

#### Carcinogenic risk (CR_i_)

Carcinogenic risk (CRi) was evaluated to find the potential risk related to the exposure to carcinogenic elements during the lifetime after the exposure (Rouhani et al., 2022).

7 $$CR_{i } = ADD_{i} \times SF$$

where SF—cancer slope factor (kg/day/mg).

For the interpretation of the results, it was adopted that CR values less than or equal to 1.00E−06 represent virtual safety, and a CR equal to or greater than 1.00E−04 indicates a potentially great risk. The range of acceptable total risk is 1.00E−06 to 1.00E−04 (Jaffar et al., 2017).

#### Incremental lifetime cancer risk (ILCR)

8 $$ILCR = \mathop \sum \limits_{i = 1}^{n} CR_{i}$$

For the interpretation of *CR*_*i*_ and *ILCR* values for each route of exposure, it was considered that a negligible carcinogenic risk occurs when ILCR < 1.00E−06, and lifetime carcinogenic risk to human health occurs when ILCR > 1.00E−04.

## Results and discussion

### Physicochemical parameters of soils and overview of HMs concentrations

**Fig. 2:**
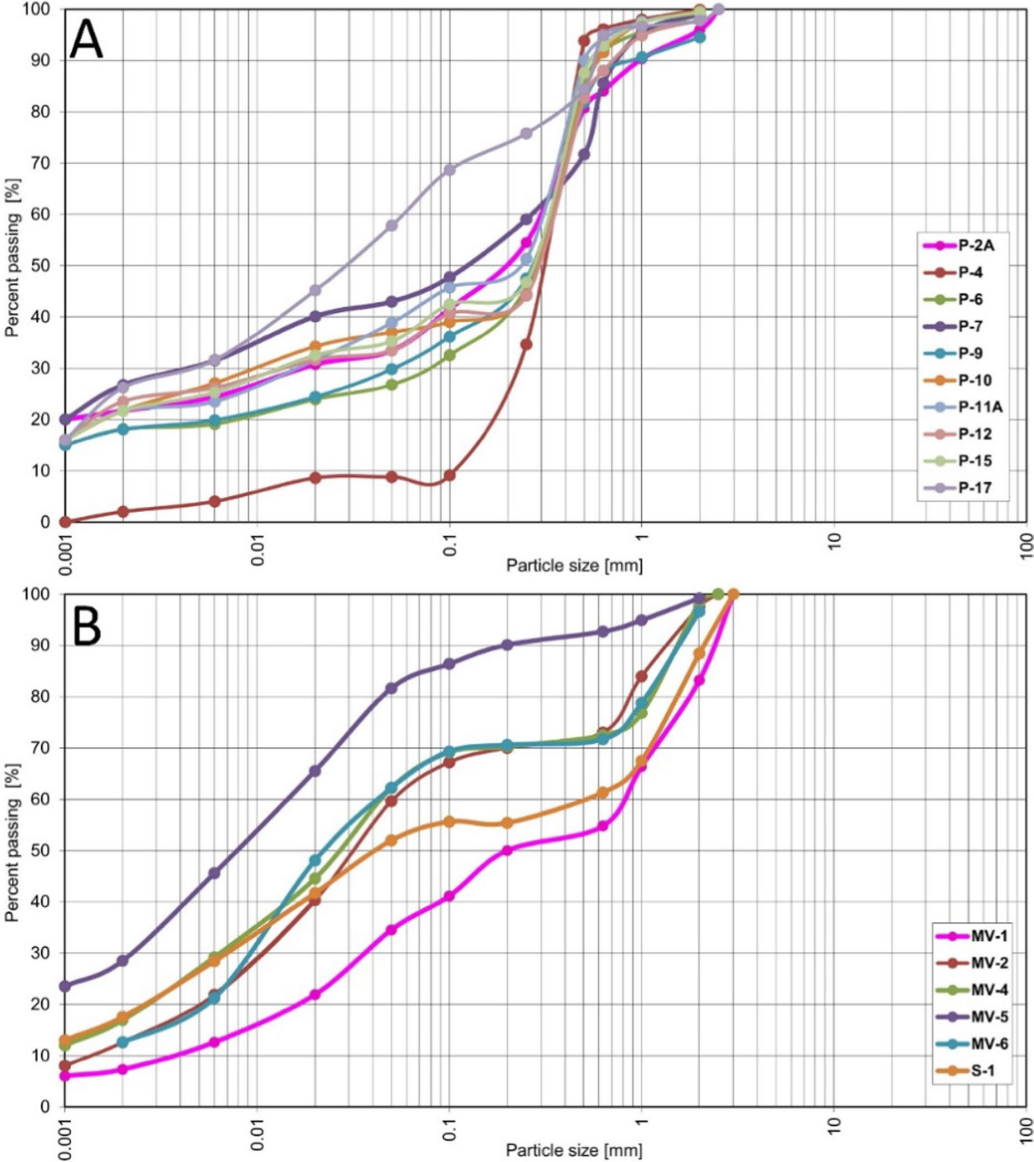
Particle size distribution of soils from the Radiowo (**A**) and Zdounky (**B**) areas

The soils collected from the Radiowo landfill area exhibited varied granulometric compositions, including sandy clay, silty clay, clays, and sand. The average distribution of the fractions in these soils was approximately 20.13% clay, 14.27% silt, 63.84% sand, and 1.76% gravel. The soils from the Zdounky area were classified as sandy clayey silts, silty clays, and clayey sands, with a specific fraction of 15.90% clay, 42.79% silt, 35.21% sand, and 6.10% gravel. The distribution of fractions in these soils is of particular importance for the possible retardation of HMs, such as Pb and Cd. The particle size distribution curves for the analyzed soils are shown in Fig. 2.

The results, as shown in the Supplementary materials (Table S6), indicate that there were no significant differences in the content of clay (*p* = 0.115779) and gravel (*p* = 0.07350) fractions between the soils from both landfills. However, significant differences were observed for the silt (*p* = 0.001993) and sand (*p* = 0.000350) fractions, as determined by the Mann–Whitney U and Student-t tests, respectively.

The soils from the Radiowo site exhibited a pH range of strongly acidic (pH = 5.0) to moderately alkaline (pH = 8.0), with a mean pH value of 7.3, indicating a neutral character (Table 1).

**Table 1:** Statistical data of pH, EC and HMS concentrations in soils (mg/kg DM) of the Radiowo and Zdounky areas

Statistical parameter	pH (–)	EC (μS/cm)	Heavy metals (mg/kg DM)				
			Ni	Cd	Pb	Zn	Cu
*Radiowo*							
Mean	7.3	293.10	4.50	3.49	28.32	52.74	12.14
Median	7.4	285.10	4.59	1.60	18.99	46.32	10.09
Minimum	5.0	26.40	0.10	0.66	6.02	16.33	2.93
Maximum	8.0	589.20	7.81	12.40	104.83	127.46	21.69
SD	0.8	152.01	2.28	4.29	29.10	31.12	6.47
Variance	0.6	23,110.38	5.20	18.36	846.62	968.60	41.81
CV	10.4	51.87	50.73	122.82	102.76	59.00	53.27
Skewness	− 2.3	0.23	− 0.47	1.78	2.38	1.63	0.42
Kurtosis	4.9	− 0.54	0.05	1.60	6.27	3.61	− 1.16
*Zdounky*							
Mean	7.4	267.74	4.73	0.10	0.10	32.05	14.73
Median	7.4	254.50	4.85	0.10	0.10	34.95	15.50
Minimum	7.2	200.80	2.40	0.10	0.10	5.20	2.70
Maximum	7.5	367.70	7.10	0.10	0.10	48.70	25.90
SD	0.1	65.08	2.28	0.00	0.00	16.62	7.60
Variance	0.01	4235.06	5.21	0.00	0.00	276.14	57.77
CV	1.5	24.31	48.23	0.00	0.00	51.85	51.59
Skewness	− 0.8	0.78	− 0.02	n.a	n.a	− 0.80	− 0.24
Kurtosis	0.3	− 0.98	− 3.15	n.a	n.a	− 0.23	1.43

*SD* standard deviation, *CV* coefficient of variation, *n.a.* not applicable

The soils from the Zdounky landfill had a pH range from neutral (pH = 7.2) to mildly alkaline (pH = 7.5), which aligns with the optimal pH range for crops.

Statistical analysis revealed no significant differences in the soil pH between the two landfills. The concentrations of most analyzed HMs in the soils did not follow a normal distribution, except for Cu. This lack of normality in HMs concentrations is consistent with Karimian et al. (2021), who reported non-normal distributions of HMs. The relatively neutral to alkaline pH values observed in the analyzed soils suggest a potentially lower mobility and reduced environmental risk associated with HMs contamination in both landfill areas.

For both landfills, the soils were found to be non-saline (EC < 2000 μS/cm) (Table 1) and the differences between measured EC values were not significant (*p* = 0.682441) (Table S6). Similar observations were presented by Vijayalakshmi et al. (2020), who measured EC in the top layers (0.00–0.30 m) in the area of landfills at levels 148–304 μS/cm. A substantially broader range (510–3655 μS/cm) of the EC at the landfill site in Ghana was observed by Obiri-Nyarko et al. (2021).

Typical EC values for agricultural soils in Poland are reported to be around 96.4 μS/cm on average. The range of these values spans from a minimum of < 10 μS/cm to a maximum of 367.4 μS/cm (Kartanowicz et al., 2022).

Regarding the conditions of the Czech Republic, Lukas et al. (2018) conducted experimental work on eight fields within the Rostenice a.s. farm enterprise, spanning a total acreage of 476 ha. Their study specifically aimed to explore the relationship between soil EC and various physico-chemical properties of the soil. The findings of their investigation revealed average EC values for agricultural soils in the range of 255.8 to 753.2 μS/cm. This study aligns with our findings and contributes to the broader understanding of the variability in soil EC across agricultural landscapes. For comparison, the results from agricultural regions in southern Spain indicate EC values averaging around 210 μS/cm. Notably, this value is reflective of areas engaged in intensive agriculture practices, as well as abandoned farmlands (Lucas-Borja et al., 2019). The results obtained regarding the EC of soils in the vicinity of the Radiowo landfill and the Zdounky landfill, surrounded by agriculturally used areas, are not extremely low. Instead, they fall within the typical ranges described in the literature.

The EC values in soils by our study were on average equal to 293.10 μS/cm for the Radiowo landfill in Poland and 267.74 μS/cm for the Zdounky landfill in the Czech Republic. The EC values in the vicinity of the Ain-El-Hammam landfill site in Algeria were reported to range from 2.0 to 2.8 μS/cm (Mouhoun-Chouaki et al., 2019). These relatively low values suggest minimal salinity in the surrounding area, indicating a lack of significant impact on soil conductivity. In the context of abandoned dump sites in Kumasi, Ghana, the average EC values were approximately 335 μS/cm, with an average pH of 7.8 (Akanchise et al., 2020). The EC values indicating the non-saline character of soil at landfill sites may be also confirmed by the climate characteristic, as the saline soils may be observed mainly under arid or semiarid climates (Yahaya et al., 2009).

The measured concentrations of Ni in the soils from both landfills did not show significant differences (Table S6). However, it is important to note that these Ni concentrations were below the typical concentrations reported by Plant and Raiswell (1983) (Ni = 5–500 mg/kg DM) and Sposito (1989) (Ni = 19 mg/kg DM) for soils.

The average concentrations of Cd (3.49 mg/kg DM) (Table 1) in the topsoil of the Radiowo area exceeded the limits set for residential and recreational areas, as well as arable areas (except for type II-3). The observed Cd concentrations were in line with the standards required for wastelands and industrial areas. Considering the limits for Cd in soils, the concentrations in Radiowo exceed seven times (in comparison to the Czech and Danish standards) and even eight times (in comparison to the Swedish standards).

Moreover, high CV values of Cd and Pb in the Radiowo area suggest that the occurrence of these HMs is affected by anthropogenic activities. Higher CV values indicate a greater variability or dispersion in the concentrations of Cd and Pb within the studied area. Conversely, low CV values are typically associated with the natural origin of HMs in soil (Wieczorek et al., 2018). It was also confirmed by Karimian et al. (2021) that high CV values of HMs could be the result of external factors (i.e. human activities). Raising awareness of the presence of even minor increments of Cd in soils remains a matter of utmost importance when formulating necessary remediation strategies (Zhou et al., 2022). It is particularly important as Cd is recognized as one of the most environmentally toxic HMs, known to exert highly detrimental effects on soil health, biological activity, plant metabolism, as well as the overall well-being of humans and animals (Ihedioha et al, 2017).

The excessive concentrations of Cd, Zn, Pb, and Cu in the vicinity of the P-10 point near the Radiowo landfill indicate the presence of a potential source of HMs pollution in that area. The elevated levels of these HMs could be attributed to nearby metallurgical production activities, which can contaminate the surrounding environment, including soil. The study conducted by Wieczorek et al. (2018) supports the notion that the excessive concentrations of Cd and Pb in soils can be attributed to the influence of mining and metallurgical activities, which involve the processing of metals. The presence of a high level of Cd measured at P-11 point, which is located in the area of a waste treatment plant, suggests a potential source of pollution related to the operation of this facility.

For the Zdounky, the concentrations of Cd at each of the monitoring points were below the detection limit of 0.10 mg/kg DM (Table 1). This suggests that the Zdounky landfill should not be considered a potential source of Cd contamination in soils.

For both sites, the average Pb concentrations were substantially lower than the environmental limits (Tables S4 and S5). For the Zdounky landfill, similar to Cd, the concentrations of Pb at each monitoring point were lower than the detection limit (0.10 mg/kg DM) (Table 1) and therefore also below the range observed in typical soils (2–300 mg/kg DM) (Plant & Raiswell, 1983).

The average Zn concentrations did not exceed the standards set by other countries (Table S5). For the Zdounky landfill, the average concentration of Zn (32.05 mg/kg DM) was more than three times lower than the limit set in the Czech regulation, and also lower than the typical concentration of Zn in soils (60 mg/kg DM) (Sposito, 1989).

The Cu concentrations at both landfill sites were similar (*p* = 0.477925) and did not exceed the limit concentrations assigned to the I-IV soil groups. Moreover, these Cu concentrations were consistent with the ranges observed in typical soils (2–100 mg/kg DM) (Hazelton & Murphy, 2016).

For the Zdounky area, the highest concentrations were observed on the eastern side, particularly near agricultural areas. This finding suggests a potential association between agricultural activities (application of fertilizers and pesticides containing HMs) and elevated levels of HMs in the soil. According to Obiri-Nyarko et al. (2021), variations of HMs may be a consequence of various sources of HMs.

Based on the above findings, it may be concluded that HMs (except for Cd in the Radiowo landfill) pose a low environmental risk (Vijayalakshmi et al., 2020). Moreover, there is no evidence to suggest that leachate migration occurs which would be confirmed by the low presence of HMs (Pb, Cu, Mn, and Cd) in soil samples (Ishchenko, 2017).

Considering HMs concentrations in soils in the tested areas, it was found that they were in the order Zn > Pb > Cu > Ni > Cd for the Radiowo landfill, and Zn > Cu > Ni > Pb = Cd for the Zdounky landfill. In comparison, in soils near a municipal waste disposal site in Silchar, Assam (India), the concentrations of HMs were observed in order Zn > Fe > Ni > Cu > Cr (Choudhury et al., 2022). The HMs in the soils around the closed Lumberstewart landfill in Bulawayo (Zimbabwe) followed the order Fe > Zn > Cu > Cr > Ni > Cd (Makuleke & Ngole-Jeme, 2020). Mavakala et al. (2022) who tested wild solid waste dumpsites in the Democratic Republic of the Congo, found the HMs in soils occurrence in the descending order: Zn > Pb > Cu > Cr > Co > Cd > As > Hg, while in study performed by Karimian et al. (2021), the sequence of HMs in landfill sampling points was Al > Fe > Mn > Cr > Cu > Pb > Ni > Co > As > Cd. These comparisons highlight the variability in HMs concentrations, influenced by site-specific factors, such as waste composition, management practices, and environmental conditions.

### Interrelationships between HMs in soils

For the Radiowo landfill, a very strong correlation was found between Pb and Zn (r = 0.95), while a strong correlation was found between Cd and Pb (r = 0.72), and between Zn and Cu (r = 0.82). Moderate correlations were observed between Cd and Zn (r = 0.69), and Pb and Cu (r = 0.65). Mentioned correlations were statistically significant (*p* < 0.05). Strong positive correlations between selected HMs may indicate their identical origins (Rouhani et al., 2022).

For the Zdounky landfill, a statistically significant (*p* < 0.05) strong correlation was observed between Ni and Zn (r = 0.87). The correlations between Ni and Cu (r = 0.77) and Zn and Cu (r = 0.78) were strong but not statistically significant. The correlation for Cd and Pb was excluded because their concentrations were below the detection limit (< 0.1 mg/kg DM). Kujawska and Cel (2019) found that positive correlations between HMs may suggest their interdependence, similar behaviour, and shared sources.

Additionally, the composition of specific soil fractions can influence HMs concentration. Xiao et al. (2019) found that the overall HMs concentration exhibited a positive correlation with clay content. For the Radiowo landfill, a moderate positive correlation (r = 0.68) was found between Cu and the silt fraction. This finding also aligns with Xiao et al. (2019), who reported a moderate correlation (r = 0.50) between Cu and the silt fraction. For the Zdounky landfill, no statistically significant correlations were observed between soil fractions and HMs. The results of the correlation matrix between all parameters analyzed in our study are summarized in Supplementary materials (Tables S7 and S8).

The PCA performed for the Radiowo landfill converted the soil parameters into three principal components (PCs), accounting for 87.12% of the total variance (Table 2).

**Table 2:** Principle component analysis for the Radiowo area

Variables	PC1	PC2	PC3
pH	0.83***	0.24	0.36*
EC	0.85***	0.26	− 0.31*
Ni	0.47*	0.65**	0.57**
Cd	0.44*	− 0.67**	0.37*
Pb	0.57**	− 0.74**	0.21
Zn	0.74**	− 0.58**	0.18
Cu	0.89***	− 0.14	0.03
Clay	0.83***	0.28	− 0.04
Silt	0.74**	− 0.06	− 0.65**
Sand	− 0.89***	− 0.23	0.33*
Gravel	0.20	0.76***	0.25
Eigenvalues	5.59	2.63	1.36
% of variance	50.81	23.90	12.41
Cumulative %	50.81	74.71	87.12

*Significant factor loading is boldfaced (***strong > 0.75; **medium 0.50–0.75; *weak 0.50–0.30) (Liu et al., 2003). The underlined values are statistically significant

PC1 accounted for 50.81% of the total variance and showed strong positive loadings for pH, EC, Cu, and clay fraction. Strong negative loading of PC1 was assigned to the sand fraction. PC2 accounted for 23.90% of the total variance and was strongly related to the gravel fraction. PC3 accounted for 12.41% of the total variance and showed a medium positive loading of Ni and a medium negative loading of the silt fraction. Strong and medium loadings of Pb, Zn, and Cu assigned to PC1 suggest the same origin of these HMs. A similar observation was reported by Obiri-Nyarko et al. (2021), who found strong positive correlations among Cu, Pb, and Zn, suggesting that these elements are highly associated with each other and may be common constituents of materials deposited in landfills Wieczorek et al. (2018), indicated that fractions of sand, silt, and clay as well as the content of Pb and Cd, are the most frequent components of PC1. This is consistent with our study (except for Cd, which is an important component of PC2).

The results of the PCA for the Zdounky landfill revealed three principal components (PCs), responsible for 95.16% of the total variance (Table 3).

**Table 3:** Principle component analysis for the Zdounky area

Variables	PC1	PC2	PC3
pH	− 0.77***	− 0.46*	− 0.32*
EC	0.60**	0.66**	0.44*
Ni	0.95***	0.22	− 0.21
Zn	0.71**	0.22	− 0.62**
Cu	0.61**	0.47*	− 0.52**
Clay	0.66**	− 0.64**	− 0.28
Silt	0.79***	− 0.40*	0.40*
Sand	− 0.67**	0.73**	0.06
Gravel	− 0.89***	0.19	− 0.39*
Eigenvalues	5.03	2.13	1.40
% of variance	55.92	23.67	15.57
Cumulative %	55.92	79.60	95.16

*Significant factor loading is boldfaced (***strong > 0.75; **medium 0.50–0.75; *weak 0.50–0.30) (Liu et al., 2003). The underlined values are statistically significant

PC1 accounted for 55.92% of the total variance, which exhibited strong negative loadings for the pH and gravel fraction. Positive loadings of PC1 were assigned to EC, Ni, Zn, Cu, silt, and fractions. PC2 accounted for 23.67% of the total variance and had a medium positive loading of EC and sand fraction. A medium negative loading of PC2 was observed concerning the clay fraction. PC3 had medium negative loadings of Zn and Cu and accounted for 15.57% of the total variance. The strong and medium impacts of HMs on PC1 may suggest their similar sources and properties (Afolagboye et al., 2020). Zhou et al. (2023), who studied the distribution of HMs in soils around the Shannan landfill site in Tibet, also used PCA to identify the sources of these elements. They found that the cumulative contribution of all factors was 69.78% and that PC1 was mostly associated with HMs (Cu, Zn, Pb, Cr, Ni, and Cd). This strong association can be attributed to the shared origin of these HMs, which was related to the specific composition of the waste present in the landfill site. Specific waste materials, such as batteries, waste tires, ink, and plastics, likely contribute to the presence and co-occurrence of these HMs in the landfill environment.

### Health risk

The results of the calculation of the average daily intake of HMs via soil ingestion, inhalation, and dermal absorption, obtained for each sampling point at the Radiowo and Zdounky sites are presented in Supplementary materials (Tables S9, Table S10, and Table S11). For the Radiowo landfill, the average HQ values for ingestion of Ni, Cd, Pb, Zn, and Cu were 3.08E−04, 4.78E−03, 1.1E−02, 2.41E−04, respectively, whereas for inhalation were: 4.53E−08, 7.03E−07, 1.63E−06, 3.54E−08, and 6.11E−08, respectively, and for dermal absorption were 3.07E−05, 1.91E−03, 2.92E−04, 9.61E−07, and 1.66E−06, respectively. This revealed that the exposure pathways of HMs in the Radiowo area occurred in the following order: ingestion > dermal absorption > inhalation (Table S12).

For the Zdounky landfill, the average HQ values for ingestion of Ni, Cd, Pb, Zn, and Cu were 3.24E−04, 1.37E−04, 3.92E−05, 1.49E−04, and 5.04E−04, respectively, while for inhalation were 4.77E−08, 2.01E−08, 5.76E−09, 2.15E−08, and 7.42E−08, respectively; and for dermal absorption were: 3.23E−05, 5.47E−05, 1.03E−06, 5.84E−07, and 2.01E−06, respectively. The exposure pathways of HMs in the Zdounky area followed the sequence: ingestion > dermal absorption > inhalation (Table S13).

For both analyzed landfills, the HQ values were lower than 1, which indicates that no potential negative effects exist (Wieczorek et al., 2018). The exposure pathways of HMs vary at different sites. While in the Zdounky and Radiowo areas, the sequence was ingestion > dermal absorption > inhalation, Wang et al. (2020) found that in MSW landfill sites, the order was inhalation > dermal absorption > ingestion. By contrast, Wang et al. (2020), Rouhani et al. (2022) found that inhalation of HMs was almost negligible compared to other exposure ways. Olagunju et al. (2020), based on their research performed in the area of Awotan Landfill in Ibadan (Southwest Nigeria), also found that inhalation of soil does not pose health hazards due to HMs. Similar to our study, Obiri-Nyarko et al. (2021) and Zhou et al. (2022) found that direct ingestion is a major pathway for exposing humans to HMs at landfill sites.

**Fig. 3:**
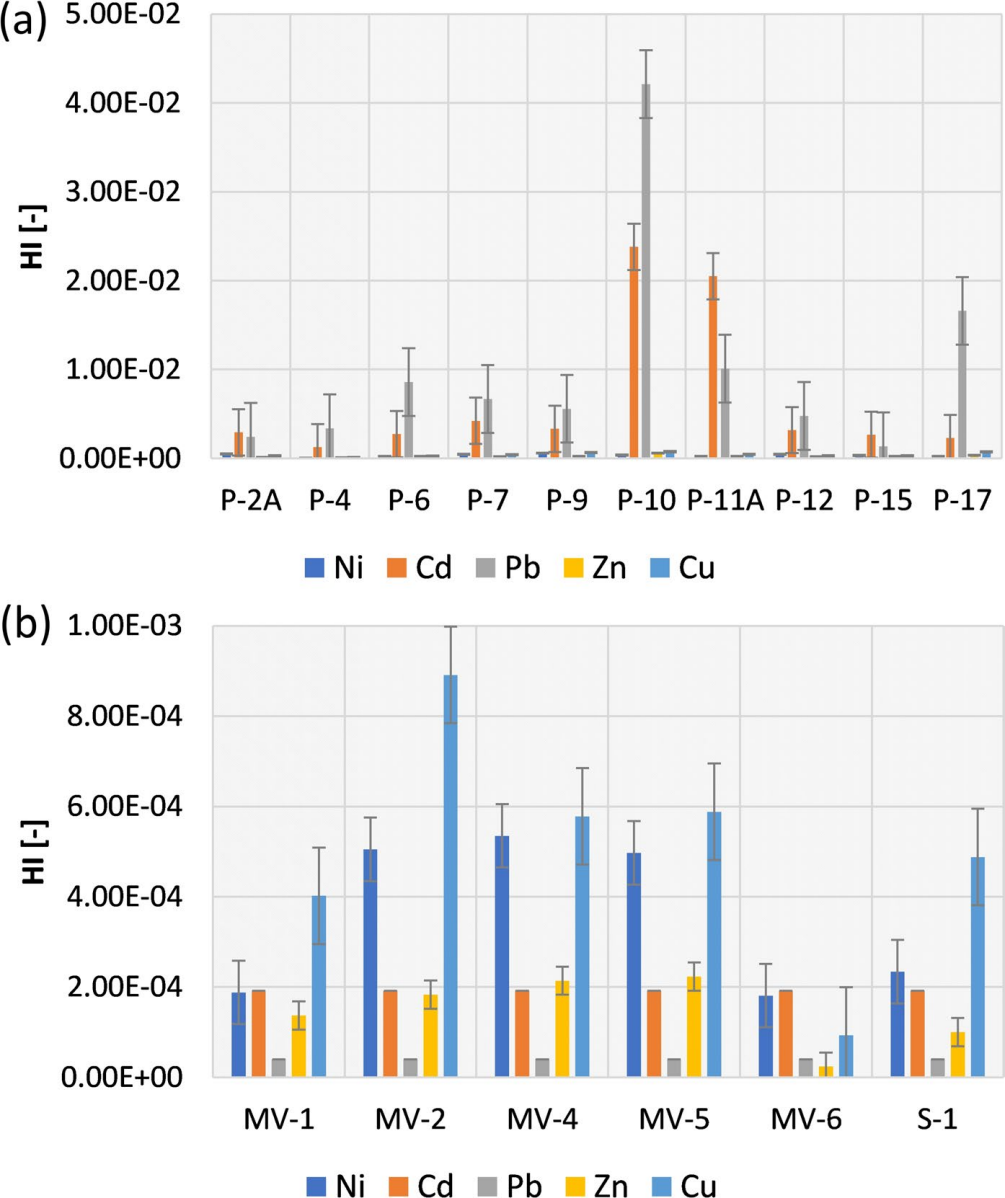
HI values for the Radiowo (**a**) and Zdounky (**b**) landfills

In terms of HI (Fig. 3), it was found that no adverse human health effects occur (HI < 1).

Rouhani et al. (2022) also revealed that the HQ and HI values for all HMs found in soils were lower than 1, indicating no non-carcinogenic risk from these elements. In contrast to these findings, Obiri-Nyarko et al. (2021) reported HI values equal to 1.72, indicating the presence of health risks associated with landfills. Although no adverse effects on human health were observed in the Radiowo and Zdounky areas, it is important to highlight the significance of taking precautions to minimize potential exposure to soils contaminated by HMs. To reduce health risks, it is highly recommended to use personal protective equipment and follow hygienic operation practices (Thongyuan et al., 2021).

For the Radiowo landfill, it was found that the average carcinogenic risks related to HMs ingestion (i.e. ingestion of contaminated food or water) were as follows: CR = 4.49E−06 for Ni, CR = 7.78E−07 for Cd, and 1.41E−07 for Pb. These values indicate that in terms of Cd and Pb, the carcinogenic risk from soil to human health is negligible (CR < 1E−06) (Table S14), whereas in the case of Ni, the risk is acceptable (1E−06 < CR < 1E−04) (Sun & Chen, 2018).

**Fig. 4:**
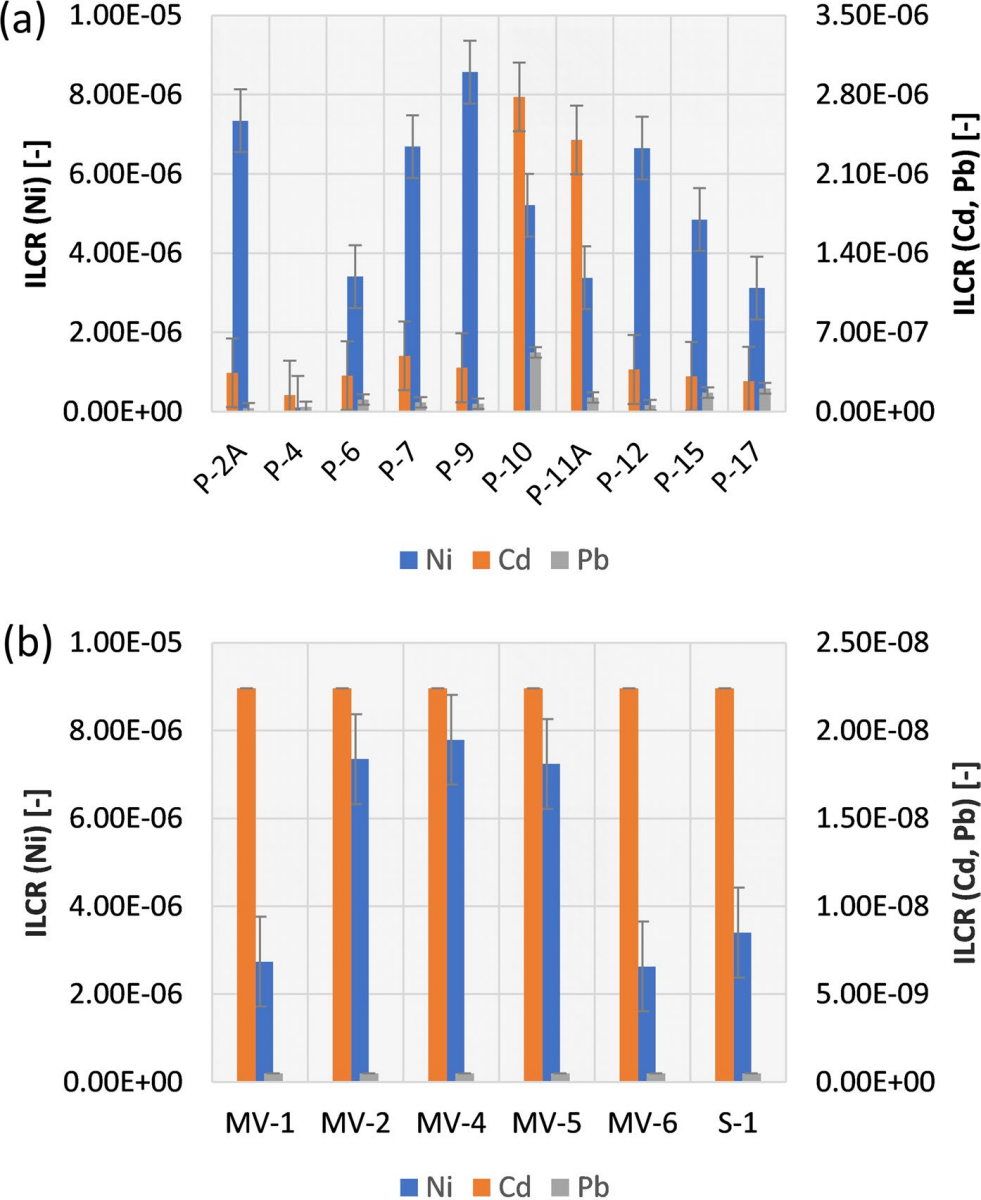
ILCR for the Radiowo and Zdounky landfills

For possible exposure by inhalation (i.e. contact with airborne particles), it was calculated that CR = 3.49E−10 for Ni, CR = 1.90E−09 for Cd and 1.02E−10 for Pb. Regarding exposure by dermal absorption (i.e. contact of contaminated soil through the skin), it was found for the Radiowo landfill area that CR = 4.48E−07 for Ni, CR = 3.11E−09 for Cd and CR = 5.64E−10 for Pb. For the analyzed exposure pathways of digestion and dermal absorption from landfill soils, it was determined that the carcinogenic risk associated with the studied HMs was negligible. This implies that the levels of exposure to HMs through these pathways have insignificant potential to cause cancer in individuals. Considering the lifetime carcinogenic risk to human health in the Radiowo landfill area, it was revealed that the ILCR values followed the sequence: ILCR = 4.93E−06 for Ni > ILCR = 7.83E−07 for Cd > ILCR = 1.42E−07 for Pb (Fig. 4a). These values also indicate negligible or acceptable carcinogenic risks caused by these HMs.

For the Zdounky landfill, the investigation of carcinogenic risk revealed that for the ingestion exposure, the average indicators were as follows: CR = 4.72E−06 for Ni, CR = 2.23E−08 for Cd, and CR = 4.99E−10 for Pb. In this case, similar to the Radiowo area, the risk of Ni was found to be acceptable and negligible for Cd and Pb. For the calculations performed for inhalation exposure, it was found that CR = 3.67E−10 for Ni, CR = 5.44E−11 for Cd, and CR = 3.63E−13 for Pb. The risk related to dermal absorption was quantified as: CR = 4.72E−07 for Ni, CR = 8.90 E−11 for Cd, and CR = 1.99E−12 for Pb. The carcinogenic risk from the soil of the Zdounky area was found to be negligible for the inhalation and dermal absorption exposure paths (Table S15).

The estimated lifetime carcinogenic risks for the landfill site were as follows: ILCR = 5.19E−06 for Ni > ILCR = 2.24E−08 for Cd > ILCR = 5.01E−10 for Pb (the same pattern was observed in the Radiowo case) (Fig. 4b).

By analyzing the carcinogenic risk, we aimed to quantify the likelihood of cancer occurrence and determine whether the exposure pathways from landfill soils pose a significant health concern. The results may be crucial for the management and remediation of landfill areas and may help in the implementation of protective measures to reduce potential health risks in tested landfill sites and other landfills of the same type.

## Conclusions

Through a comparative analysis of pollution patterns, as well as human health risk assessment, this study provides valuable insights into the variations in HMs occurrence, exposure pathways, sources, and potential risks across different MSW landfill sites. These findings contribute to the understanding of the site-specific factors that influence possible contamination levels and associated hazards. The findings of this study, supported by an analysis of literature data from a global perspective, can contribute to the understanding of the specific challenges posed by MSW landfills. Comparative analysis provides insights into the similarities and differences in risk profiles between two different landfill sites classified as sanitary landfills. The identification of heavy Cd pollution in the Radiowo area highlights the importance of identifying this specific contaminant. The geostatistical analysis provides new premises for preliminary site recognition before detailed sampling campaigns at landfill sites. The assessment of human health risks, including exposure pathways, Hazard Quotient (HQ), Hazard Index (HI), and Incremental Lifetime Cancer Risk (ILCR), provides valuable information on potential health impacts. The findings of this study suggest that merely tracking the range of HMs concentrations may not be sufficient for comprehensive risk assessment purposes. These results indicate that elevated concentrations of certain HMs, such as Cd in the Radiowo area or Cu in the Zdounky area, do not necessarily signify immediate adverse health effects. This observation highlights the importance of conducting a thorough risk assessment that considers various factors beyond the concentration levels.

### Supplementary Information

Below is the link to the electronic supplementary material.

Supplementary file1 (DOCX 66 KB)

## References

[CR1] Adegboye MA, Adekanmi AA, Lawal IK, Owolabi OA, Omole FO (2021). Evaluation of heavy metals in soils from different dumpsites. Evaluation.

[CR2] Aendo P, Netvichian R, Thiendedsakul P, Khaodhiar S, Tulayakul P (2022). Carcinogenic risk of Pb, Cd, Ni, and Cr and critical ecological risk of Cd and Cu in soil and groundwater around the municipal solid waste open dump in central Thailand. Journal of Environmental and Public Health.

[CR3] Afolagboye LO, Ojo AA, Talabi AO (2020). Evaluation of soil contamination status around a municipal waste dumpsite using contamination indices, soil-quality guidelines, and multivariate statistical analysis. SN Applied Sciences.

[CR4] Akanchise T, Boakye S, Borquaye LS, Dodd M, Darko G (2020). Distribution of heavy metals in soils from abandoned dump sites in Kumasi, Ghana. Scientific African.

[CR5] Ali H, Khan E, Ilahi I (2019). Environmental chemistry and ecotoxicology of hazardous heavy metals: Environmental persistence, toxicity, and bioaccumulation. Journal of Chemistry.

[CR6] Ali IH, Siddeeg SM, Idris AM, Brima EI, Ibrahim KA, Ebraheem SA, Arshad M (2021). Contamination and human health risk assessment of heavy metals in soil of a municipal solid waste dumpsite in Khamees-Mushait, Saudi Arabia. Toxin Reviews.

[CR7] Barlaz MA, Rooker AP, Kjeldsen P, Gabr MA, Borden RC (2002). Critical evaluation of factors required to terminate the postclosure monitoring period at solid waste landfills. Environmental Science and Technology.

[CR8] Bruce, R. C., & Rayment, G. E. (1982). *Analytical methods and interpretations used by the Agricultural Chemistry Branch for Soil and Land Use Surveys*. Queensland Department of Primary Industries. Bulletin QB8 (2004), Indooroopilly, Queensland.

[CR9] Choudhury M, Jyethi DS, Dutta J, Purkayastha SP, Deb D, Das R, Roy G, Sen T, Bhattacharyya KG (2022). Investigation of groundwater and soil quality near to a municipal waste disposal site in Silchar, Assam, India. International Journal of Energy and Water Resources.

[CR10] Debnárová A, Weissmannová H (2010). Assessment of heavy metal pollution (Cd, Cu, Pb, Hg) in urban soils of roadsides in Brno. Transactions on Transport Sciences.

[CR11] Decree No. 153/2016 Coll., on determining the details of agricultural soil quality protection for ordinary soils. Available online: https://www.zakonyprolidi.cz/cs/2016-153. Accessed 20 May 2023.

[CR12] Draghici, C., Jelescu, C., Dima, C., Coman, G., & Chirila, E. (2011). Heavy metals determination in environmental and biological samples. In *Environmental heavy metal pollution and effects on child mental development: Risk assessment and prevention strategies* (pp. 145–158). Springer Netherlands.

[CR14] El Fadili H, Ali MB, Touach N, El Mahi M (2022). Ecotoxicological and pre-remedial risk assessment of heavy metals in municipal solid wastes dumpsite impacted soil in Morocco. Environmental Nanotechnology, Monitoring & Management.

[CR15] Emenike EC, Iwuozor KO, Anidiobi SU (2021). Heavy metal pollution in aquaculture: Sources, impacts and mitigation techniques. Biological Trace Element Research.

[CR16] Farrukh MA (2012). Atomic absorption spectroscopy.

[CR201] Fronczyk, J., Sieczka, A., Lech, M., Radziemska, M., & Lechowicz, Z. (2016). Transport of Nitrogen Compounds through Subsoils in Agricultural Areas: Column Tests. *Polish Journal of Environmental Studies,**25*(4), 1505–1514. 10.15244/pjoes/62340

[CR17] Hazelton P, Murphy B (2016). Interpreting soil test results: What do all the numbers mean?.

[CR18] He R, Sandoval-Reyes M, Scott I, Semeano R, Ferrao P, Matthews S, Small MJ (2022). Global knowledge base for municipal solid waste management: Framework development and application in waste generation prediction. Journal of Cleaner Production.

[CR19] Hussein M, Yoneda K, Mohd-Zaki Z, Amir A, Othman N (2021). Heavy metals in leachate, impacted soils and natural soils of different landfills in Malaysia: An alarming threat. Chemosphere.

[CR20] Ihedioha JN, Ukoha PO, Ekere NR (2017). Ecological and human health risk assessment of heavy metal contamination in soil of a municipal solid waste dump in Uyo, Nigeria. Environmental Geochemistry and Health.

[CR21] Iravanian, A., & Ravari, S. O. (2020). Types of contamination in landfills and effects on the environment: A review study. In *IOP conference series: Earth and environmental science* (vol. 614, no. 1, p. 012083). IOP Publishing.

[CR22] Ishchenko V (2017). Soil contamination by heavy metal mobile forms near landfills. International Journal of Environment and Waste Management.

[CR23] Jaffar STA, Luo F, Ye R, Younas H, Hu XF, Chen LZ (2017). The extent of heavy metal pollution and their potential health risk in topsoils of the massively urbanized district of Shanghai. Archives of Environmental Contamination and Toxicology.

[CR24] Jakimiuk A (2022). Review of technical methods landfill sealing and reclamation in the world. Acta Scientiarum Polonorum Architectura.

[CR25] Jakimiuk A, Matsui Y, Podlasek A, Vaverková MD (2022). Assessment of landfill protection systems in Japan—A case study. Acta Scientiarum Polonorum. Architectura.

[CR26] Kaiser HF (1958). The varimax criterion for analytic rotation in factor analysis. Psychometrika.

[CR27] Karimian S, Shekoohiyan S, Moussavi G (2021). Health and ecological risk assessment and simulation of heavy metal-contaminated soil of Tehran landfill. RSC Advances.

[CR28] Kartanowicz, R., Stefaniak, M., Mederska-Mazur, A., Radosz, Ł., Stanek, K., Stanek, A., Mutwil, D. (2022). *Raport z III etapu realizacji zamówienia “Monitoring chemizmu gleb ornych w Polsce w latach 2020–2022”*. Eurofins OBiKŚ Sp. z.o.o., Katowice, Poland (in Polish). Available online: https://www.gios.gov.pl/images/dokumenty/pms/monitoring_jakosci_gleb/raport_chemizm_gleb_2022.pdf. Accessed 22 December 2023

[CR200] Kicińska, A., & Wikar, J. (2021). Ecological risk associated with agricultural production in soils contaminated by the activities of the metal ore mining and processing industry-example from southern Poland. *Soil and Tillage Research,**205*, 104817.

[CR29] Koda E (2012). Influence of vertical barrier surrounding old sanitary landfill on eliminating transport of pollutants on the basis of numerical modelling and monitoring results. Polish Journal of Environmental Studies..

[CR30] Koda E, Sieczka A, Osinski P (2016). Ammonium concentration and migration in groundwater in the vicinity of waste management site located in the neighborhood of protected areas of Warsaw. Poland. Sustainability.

[CR31] Koda E, Miszkowska A, Osinski P, Sieczka A (2020). Heavy metals contamination within restored landfill site in Poland. Environmental Geotechnics..

[CR32] Koda E, Kiersnowska A, Kawalec J, Osiński P (2020). Landfill slope stability improvement incorporating reinforcements in reclamation process applying observational method. Applied Sciences.

[CR33] Kosowski K, Tucki K, Piwowarski M, Stępień R, Orynycz O, Włodarski W (2019). Thermodynamic cycle concepts for high-efficiency power plants. Part B: Prosumer and distributed power industry. Sustainability.

[CR34] Kujawska, J., & Cel, W. (2019). Potential ecological risk assessment and prediction of heavy-metal pollution of soil surrounding the drilling waste deposition site. In *MATEC web of conferences* (vol. 252, p. 09011). EDP Sciences, 10.1051/matecconf/201925209011

[CR35] Liu CW, Lin KH, Kuo YM (2003). Application of factor analysis in the assessment of groundwater quality in a blackfoot disease area in Taiwan. Science of the Total Environment.

[CR36] Liu P, Hu W, Tian K, Huang B, Zhao Y, Wang X, Zhou Y, Shi B, Kwon BO, Choi K, Ryu J, Chen Y, Wang T, Khim JS (2020). Accumulation and ecological risk of heavy metals in soils along the coastal areas of the Bohai Sea and the Yellow Sea: A comparative study of China and South Korea. Environment International.

[CR37] Lucas-Borja ME, Zema DA, Plaza-Álvarez PA, Zupanc V, Baartman J, Sagra J, González-Romero J, Moya D, de las Heras J (2019). Effects of different land uses (abandoned farmland, intensive agriculture and forest) on soil hydrological properties in Southern Spain. Water.

[CR38] Lukas V, Neudert L, Novák J, Kren J (2018). Estimation of soil physico-chemical properties by on-the-go measurement of soil electrical conductivity. Agriculturae Conspectus Scientificus.

[CR39] Maji KJ, Chaudhary R (2019). Principal component analysis for water quality assessment of the Ganga River in Uttar Pradesh, India. Water Resources.

[CR40] Makuleke P, Ngole-Jeme VM (2020). Soil heavy metal distribution with depth around a closed landfill and their uptake by Datura stramonium. Applied and Environmental Soil Science.

[CR41] Matheson T (2022). Disposal is not free: Fiscal instruments to internalize the environmental costs of solid waste. International Tax and Public Finance.

[CR42] Mavakala BK, Sivalingam P, Laffite A, Mulaji CK, Giuliani G, Mpiana PT, Poté J (2022). Evaluation of heavy metal content and potential ecological risks in soil samples from wild solid waste dumpsites in developing country under tropical conditions. Environmental Challenges.

[CR43] Morita AK, Ibelli-Bianco C, Anache JA, Coutinho JV, Pelinson NS, Nobrega J, Rosalem LMP, Leite CMC, Niviadonski LM, Manastella C, Wendland E (2021). Pollution threat to water and soil quality by dumpsites and non-sanitary landfills in Brazil: A review. Waste Management.

[CR44] Mouhoun-Chouaki S, Derridj A, Tazdaït D, Salah-Tazdaït R (2019). A study of the impact of municipal solid waste on some soil physicochemical properties: The case of the landfill of Ain-El-Hammam Municipality, Algeria. Applied and Environmental Soil Science.

[CR45] Nartowska, E., Kozłowski, T., & Kolankowska, M. (2017). The changes in the microstructure of ion-exchanged clays. In *E3S web of conferences* (vol. 17, p. 00063). EDP Sciences. 10.1051/e3sconf/20171700063

[CR47] Nkwunonwo UC, Odika PO, Onyia NI (2020). A review of the health implications of heavy metals in food chain in Nigeria. The Scientific World Journal.

[CR48] Obiri-Nyarko F, Duah AA, Karikari AY, Agyekum WA, Manu E, Tagoe R (2021). Assessment of heavy metal contamination in soils at the Kpone landfill site, Ghana: Implication for ecological and health risk assessment. Chemosphere.

[CR49] Olagunju TE, Olagunju AO, Akawu IH, Ugokwe CU (2020). Quantification and risk assessment of heavy metals in groundwater and soil of residential areas around Awotan landfill, Ibadan, Southwest-Nigeria. Journal of Toxicology and Risk Assessment.

[CR50] Ore OT, Adeola AO (2021). Toxic metals in oil sands: Review of human health implications, environmental impact, and potential remediation using membrane-based approach. Energy, Ecology and Environment.

[CR51] Plant JA, Raiswell R, Thornton I (1983). Principles of environmental geochemistry. Applied environmental geochemistry.

[CR52] PN-EN ISO 10390. (2022). *Soil, treated biowaste and sewage sludge—Determination of pH*. Polish Committee for Standardization (PKN), Warsaw, Poland.

[CR53] PN-EN ISO 14688–1. (2018). *Geotechnical investigation and testing—Identification and classification of soil—Part 1: Identification and description*. Polish Committee for Standardization (PKN), Warsaw, Poland.

[CR54] PN-ISO 10381–1. (2008). *Soil quality—Sampling—Part 1: Principles for developing sampling programmes*. Polish Committee for Standardization (PKN), Warsaw, Poland.

[CR55] PN-ISO 10381–2. (2007). *Soil quality—Sampling—Part 2: Principles of sampling techniques*. Polish Committee for Standardization (PKN), Warsaw, Poland.

[CR56] PN-ISO 10381–3. (2007). *Soil quality—Sampling—Part 3: Safety rules*. Polish Committee for Standardization (PKN), Warsaw, Poland.

[CR57] PN-ISO 10381–5. (2009). *Soil quality—Sampling—Part 5: Principles of practice when testing urban and industrial sites for soil contamination*. Polish Committee for Standardization (PKN), Warsaw, Poland.

[CR58] Podlasek A, Jakimiuk A, Vaverková MD, Koda E (2021). Monitoring and assessment of groundwater quality at landfill sites: Selected case studies of Poland and the Czech Republic. Sustainability.

[CR59] Podlasek A, Vaverková MD, Koda E, Jakimiuk A, Barroso PM (2023). Characteristics and pollution potential of leachate from municipal solid waste landfills: Practical examples from Poland and the Czech Republic and a comprehensive evaluation in a global context. Journal of Environmental Management.

[CR60] Priya AK, Gnanasekaran L, Dutta K, Rajendran S, Balakrishnan D, Soto-Moscoso M (2022). Biosorption of heavy metals by microorganisms: Evaluation of different underlying mechanisms. Chemosphere.

[CR61] Pysarenko P, Samojlik M, Taranenko A, Tsova Y, Horobets M, Sergii F (2022). Monitoring of municipal solid waste landfill impact on environment in Poltava Region, Ukraine. Ecological Engineering & Environmental Technology.

[CR62] Rabiej M (2018). Statistical analysis with Statistica and Excel.

[CR63] Regulation of the Minister of the Environment on the method of assessing the pollution of the Earth’s surface dated on September 1, 2016. Journal of Laws 2016, item 1395. Available online: https://isap.sejm.gov.pl/isap.nsf/DocDetails.xsp?id=wdu20160001395. Accessed 20 April 2023

[CR64] Richards, L. A. (Ed.) (1954). *Diagnosis and improvement of saline and alkaline soils*. USDA Handbook No. 60, Washington, DC.

[CR65] Rouhani A, Bradák B, Makki M, Ashtiani B, Hejcman M (2022). Ecological risk assessment and human health risk exposure of heavy metal pollution in the soil around an open landfill site in a developing country (Khesht, Iran). Arabian Journal of Geosciences.

[CR66] Schober P, Boer C, Schwarte LA (2018). Correlation coefficients: Appropriate use and interpretation. Anesthesia and Analgesia.

[CR67] Siddiqua A, Hahladakis JN, Al-Attiya WAK (2022). An overview of the environmental pollution and health effects associated with waste landfilling and open dumping. Environmental Science and Pollution Research.

[CR68] Sieczka A, Koda E (2016). Kinetic and equilibrium studies of sorption of ammonium in the soil–water environment in agricultural areas of Central Poland. Applied Sciences.

[CR69] Sposito G (1989). The chemistry of soils.

[CR70] Subba Rao N, Sunitha B, Adimalla N, Chaudhary M (2020). Quality criteria for groundwater use from a rural part of Wanaparthy District, Telangana State, India, through ionic spatial distribution (ISD), entropy water quality index (EWQI) and principal component analysis (PCA). Environmental Geochemistry and Health.

[CR71] Sun Z, Chen J (2018). Risk assessment of potentially toxic elements (PTEs) pollution at a rural industrial wasteland in an abandoned metallurgy factory in North China. International Journal of Environmental Research and Public Health.

[CR73] Thongyuan S, Khantamoon T, Aendo P, Binot A, Tulayakul P (2021). Ecological and health risk assessment, carcinogenic and non-carcinogenic effects of heavy metals contamination in the soil from municipal solid waste landfill in Central, Thailand. Human and Ecological Risk Assessment: An International Journal.

[CR74] Trach Y (2020). Metoda perspektywna usuwania metali ciężkich z wód podziemnych zachodniej Ukrainy. Acta Scientiarum Polonorum. Architectura.

[CR75] Tucki K, Orynycz O, Wasiak A, Świć A, Mieszkalski L, Wichłacz J (2020). Low emissions resulting from combustion of forest biomass in a small scale heating device. Energies.

[CR76] U.S. Environmental Protection Agency (USEPA). (2007). *Method 3051A (SW-846): Microwave assisted acid digestion of sediments, sludges, and oils*. Revision 1. Washington, DC.

[CR77] U.S. Environmental Protection Agency (USEPA). Office of Health, and Environmental Assessment. Exposure Assessment Group. (1989). *Exposure factors handbook* (vol. 90, no. 106774). Office of Health and Environmental Assessment, US Environmental Protection Agency.

[CR78] Vaverková MD (2019). Impact assessment of the municipal solid landfill on the environment: A case study. Acta Scientiarum Polonorum. Architectura.

[CR79] Vaverková MD (2023). Assessment of selected landfill impacts on selected segments of the environment—A case study. Acta Scientiarum Polonorum. Architectura.

[CR80] Vijayalakshmi P, Raji PK, Eshanthini P, Bennetm RRV, Ravi R (2020). Analysis of soil characteristics near the solid waste landfill site. Nature Environment and Pollution Technology.

[CR81] Wang X, Dan Z, Cui X, Zhang R, Zhou S, Wenga T, Yan B, Chen G, Zhang Q, Zhong L (2020). Contamination, ecological and health risks of trace elements in soil of landfill and geothermal sites in Tibet. Science of the Total Environment.

[CR205] Wieczorek, J., Baran, A., Urbański, K., Mazurek, R., & Klimowicz-Pawlas, A. (2018). Assessment of the pollution and ecological risk of lead and cadmium in soils. *Environmental Geochemistry and Health,**40*, 2325-2342. 10.1007/s10653-018-0100-5 10.1007/s10653-018-0100-5 PMC6280874 29589150

[CR210] Xiao, L., Guan, D., Chen, Y., Dai, J., Ding, W., Peart, M. R., & Zhang, C. (2019). Distribution and availability of heavy metals in soils near electroplating factories. *Environmental Science and Pollution Research,**26*, 22596–22610. 10.1007/s11356-019-04706-0 10.1007/s11356-019-04706-0 31165447

[CR82] Yahaya MI, Mohammad S, Abdullahi BK (2009). Seasonal variations of heavy metals concentration in abattoir dumping site soil in Nigeria. Journal of Applied Sciences and Environmental Management.

[CR83] Yeilagi S, Rezapour S, Asadzadeh F (2021). Degradation of soil quality by the waste leachate in a Mediterranean semi-arid ecosystem. Scientific Reports.

[CR84] Zhou P, Zeng D, Wang X, Tai L, Zhou W, Zhuoma Q, Lin F (2022). Pollution levels and risk assessment of heavy metals in the soil of a landfill site: A case study in Lhasa, Tibet. International Journal of Environmental Research and Public Health.

[CR85] Zhou W, Dan Z, Meng D, Zhou P, Chang K, Zhuoma Q, Wang J, Xu F, Chen G (2023). Distribution characteristics and potential ecological risk assessment of heavy metals in soils around Shannan landfill site, Tibet. Environmental Geochemistry and Health.

