# Supplementary material for: A comprehensive investigation of geoenvironmental pollution and health effects from municipal solid waste landfills
Source: Environ Geochem Health. 2024 Feb 23;46(3):97. doi: 10.1007/s10653-024-01852-4 (PMC10891210; doi:10.1007/s10653-024-01852-4)
Supplement: Supplementary file 1 — Supplementary file1 (DOCX 66 KB) [file 10653_2024_1852_MOESM1_ESM.docx]

**A Comprehensive Investigation of Geoenvironmental Pollution and Health Effects from Municipal Solid Waste Landfills**

Anna Podlasek^a^, Magdalena Daria Vaverková^a,b,*^, Aleksandra Jakimiuk^a^, Eugeniusz Koda^a^

^a^ *Department of Revitalization and Architecture, Institute of Civil Engineering, Warsaw University of Life Sciences – SGGW, Nowoursynowska 159, 02 776, Warsaw, Poland;* [anna_podlasek@sggw.edu.pl](mailto:anna_podlasek@sggw.edu.pl), [aleksandra_jakimiuk@sggw.edu.pl](mailto:aleksandra_jakimiuk@sggw.edu.pl),

[eugeniusz_koda@sggw.edu.pl](mailto:eugeniusz_koda@sggw.edu.pl)

^b^ *Department of Applied and Landscape Ecology, Faculty of AgriSciences, Mendel University in Brno, Zemědělská 1, 613 00, Brno, Czech Republic;* [magda.vaverkova@uake.cz](mailto:magda.vaverkova@uake.cz)

^*^Corresponding author: Magdalena Daria Vaverková, [magdalena_vaverkova@sggw.edu.pl](mailto:magdalena_vaverkova@sggw.edu.pl)

Supplementary materials

Table S1. The morphology of wastes disposed at the Radiowo landfill.

| **Waste code** | **Type of waste** | **Content [%]** |
| --- | --- | --- |
| 190501 | Non-composted fraction of municipal and similar wastes | 86.96 |
| 190502 | Non-composted fraction of animal and vegetable waste | 8.69 |
| 190599 | Wastes not otherwise specified | 4.35 |

Table S2. The morphology of wastes reused for the Radiowo landfill reclamation.

| **Waste code** | **Type of waste** | **Content [%]** |
| --- | --- | --- |
| 170101 | Concrete | 3.28 |
| 170102 | Bricks | 9.83 |
| 170103 | Tiles and ceramics | 1.64 |
| 170107 | Mixtures of concrete, bricks, tiles and ceramics other than those mentioned in 17 01 06 | 6.56 |
| 170180 | Removed plaster, wallpaper, coverings | 1.64 |
| 170181 | Waste from road repairs and reconstruction | 1.64 |
| 170182 | Other wastes not specified | 1.64 |
| 170504 | Soil and stones other than those mentioned in 17 05 03 | 32.78 |
| 170506 | Dredging spoil other than those mentioned in 17 05 05 | 6.56 |
| 170508 | Track ballast other than those mentioned in 17 05 07 | 6.56 |
| 170904 | Mixed construction and demolition wastes other than those mentioned in 17 09 01, 17 09 02 and 17 09 03 | 6.56 |
| 190503 | Off-specification compost | 9.83 |
| 190999 | Wastes not otherwise specified | 1.64 |
| 200202 | Soil and stones | 6.56 |
| 200303 | Street-cleaning residues | 3.28 |

Table S3. The morphology of wastes disposed at the Zdounky landfill.

| **Waste code** | **Type of waste** | **Content [%]** |
| --- | --- | --- |
| 020104 | Waste plastics (except packaging) | 0.73 |
| 020304 | Materials unsuitable for consumption or processing | 0.15 |
| 040222 | Wastes from processed textile fibres | 0.00 |
| 070213 | Waste plastic | 1.08 |
| 100101 | Bottom ash, slag and boiler dust (excluding boiler dust mentioned in 10 01 04) | 5.63 |
| 120105 | Plastic shavings and turnings | 0.23 |
| 120121 | Spent grinding bodies and grinding materials other than those mentioned in 12 01 20 | 0.16 |
| 160119 | Plastic | 0.01 |
| 160120 | Glass | 0.01 |
| 161104 | Other linings and refractories from metallurgical processes other than those mentioned in 16 11 03 | 0.12 |
| 170101 | Concrete | 0.16 |
| 170102 | Bricks | 3.55 |
| 170107 | Mixtures of concrete, bricks, tiles and ceramics other than those mentioned in 17 01 06 | 0.08 |
| 170202 | Glass | 0.01 |
| 170203 | Plastic | 0.02 |
| 170504 | Soil and stones other than those mentioned in 17 05 03 | 4.31 |
| 170604 | Insulation materials other than those mentioned in 17 06 01 and 17 06 03 | 0.71 |
| 170904 | Mixed construction and demolition wastes other than those mentioned in 17 09 01, 17 09 02 and 17 09 03 | 1.14 |
| 190503 | Off-specification compost | 1.44 |
| 190801 | Screenings | 0.17 |
| 190802 | Waste from desanding | 0.08 |
| 191006 | Other fractions other than those mentioned in 19 10 05 | 0.79 |
| 191212 | Other wastes (including mixtures of materials) from mechanical Treatment of wastes other than those mentioned in 19 12 11 | 2.43 |
| 200111 | Textiles | 0.12 |
| 200139 | Plastics | 0.05 |
| 200203 | Other non-biodegradable wastes | 0.62 |
| 200301 | Mixed municipal waste | 44.67 |
| 200302 | Waste from markets | 0.65 |
| 200303 | Street-cleaning residues | 1.77 |
| 200307 | Bulky waste | 20.55 |
| 020304 | Materials unsuitable for consumption or processing | 0.20 |
| 200201 | Biodegradable waste | 8.37 |

Table S4. Permissible content of selected HMs, specified for a depth of 0–0.25 m below ground level for the I-IV group of soils (Own elaboration based on Journal of Laws No. 2016 item 1395).

| No. | Heavy metal | Limit concentration [mg/kg DM] | | | | | |
| --- | --- | --- | --- | --- | --- | --- | --- |
|  |  | I | II | | | III | IV |
|  |  |  | II-1 | II-2 | II-3 |  |  |
| 1 | Ni | 150 | 100 | 150 | 300 | 300 | 500 |
| 2 | Cd | 2 | 2 | 3 | 5 | 10 | 15 |
| 3 | Pb | 200 | 100 | 250 | 500 | 500 | 600 |
| 4 | Zn | 500 | 300 | 500 | 1000 | 1000 | 2000 |
| 5 | Cu | 200 | 100 | 150 | 300 | 300 | 600 |

*Notes: DM – dry matter; I – residential areas, recreational and leisure areas; II – arable areas, orchards, meadows and pastures, depending on soil features: II-1 - very light mineral soils, with the FG02 fraction less than 10%, regardless of the pH_KCl_ value, light mineral soils, with the FG02 fraction content of 10-20%, with a pH_KCl_ value less than or equal to 6.5; II-2 - light mineral soils, with the FG02 fraction content of 10-20%, with a pH_KCl_ value higher than 6.5, medium mineral soils, with the FG02 fraction content of 20-35%, with a pH_KCl_ value less than or equal to 5.5, heavy mineral soils, with the content of the FG02 fraction greater than 35%, with a pH_KCl_ value less than or equal to 5.5, mineral-organic soils with an organic carbon content of 3.5–6%, regardless of the pH_KCl_ value; II-3 - medium mineral soils, with the FG02 fraction content of 20-35%, with a pH_KCl_ value higher than 5.5, heavy mineral soils, with the content of the FG02 fraction higher than 35%, with a pH_KCl_ value higher than 5.5, mineral-organic and organic soils, with an organic carbon content of more than 6%, regardless of the value pH_KCl_; III – forests, wooded and bushy lands, wastelands, historic areas, ecological lands; IV – industrial areas, mining lands, transportation areas; FG02 - the content of the granulometric fraction in the soil with an equivalent diameter of grains below 0.02 mm.*

Table S5. Environmental standards for HMs content in soils in selected countries (Chen et al., 2018).

| Heavy metal | Countries | | | | | | | | | | | | | | | | |
| --- | --- | --- | --- | --- | --- | --- | --- | --- | --- | --- | --- | --- | --- | --- | --- | --- | --- |
|  | AU | BE | BG | CA | CH | CZ | DE | DK | FI | FR | GB | JP | NL | | NO | SE | US |
|  |  |  |  |  |  |  |  |  |  |  |  |  | A | B |  |  |  |
|  | Concentration [mg/kg DM] | | | | | | | | | | | | | | | | |
| Ni | 70 | 470 | 6 | 50 | NA | 50 | 140 | 3 | 50 | 140 | 50 | NA | 35 | 210 | 50 | 35 | 1600 |
| Cd | 2 | 6 | 0.6 | 10 | 20 | 0.5 | 20 | 0.5 | 1 | 20 | 8 | 150 | 0.8 | 12 | 3 | 0.4 | 37 |
| Pb | 100 | 700 | 40 | 140 | 1000 | 60 | 400 | 40 | 60 | 400 | 450 | 150 | 85 | 530 | 60 | 80 | 400 |
| Zn | 200 | 1000 | 110 | 200 | 2000 | 120 | NA | 500 | 200 | 9000 | NA | NA | 140 | 720 | 100 | 350 | 23000 |
| Cu | 100 | 400 | 50 | 63 | 1000 | 60 | NA | 500 | 100 | 190 | NA | NA | 36 | 190 | 100 | 100 | 3100 |

*Notes: AU – Australia, BE – Belgium, BG – Bulgaria, CA – Canada, CH – Switzerland, CZ – Czech Republic, DE – Germany, DK – Denmark, FI – Finland, FR – France, GB – United Kingdom, JP – Japan, NL – Netherlands, NO – Norway, SE – Sweden, US – United States, A – target value, B – intervention value, NA- not applicable.*

Table S6. Statistical tests of differences between selected parameters measured at the Radiowo and Zdounky sites.

| **Variables** | **Test** | **p-value** |
| --- | --- | --- |
| **Clay** | **Mann-Whitney U** | **p=0.115779, p>α** |
| Silt | Mann-Whitney U | p=0.001993, p<α |
| Sand | Student t-test | p=0.000350, p<α |
| **Gravel** | **Mann-Whitney U** | **p=0.07350, p>α** |
| **pH** | **Mann-Whitney U** | **p=0.744882, p>α** |
| **EC** | **Student t-test** | **p=0.682441, p>α** |
| **Ni** | **Mann-Whitney U** | **p=0.956750, p>α** |
| Cd | Mann-Whitney U | p=0.001376, p<α |
| Pb | Mann-Whitney U | p=0.001376, p<α |
| **Zn** | **Mann-Whitney U** | **p=0.175159, p>α** |
| **Cu** | **Student t-test** | **p=0.477925, p>α** |

*Notes: Bolded values mean that the differences between parameters are not significant.*

Table S7. Correlation matrix between soil parameters at the Radiowo landfill

| r | pH | EC | Ni | Cd | Pb | Zn | Cu | Clay | Silt | Sand | Gravel |
| --- | --- | --- | --- | --- | --- | --- | --- | --- | --- | --- | --- |
| pH | 1.00 | **0.66** | **0.74** | 0.37 | 0.33 | 0.50 | 0.62 | **0.86** | 0.33 | **-0.69** | 0.30 |
| EC | **0.66** | 1.00 | 0.39 | 0.11 | 0.18 | 0.42 | **0.76** | **0.73** | **0.78** | **-0.86** | 0.28 |
| Ni | **0.73** | 0.39 | 1.00 | -0.04 | -0.07 | 0.07 | 0.32 | 0.58 | -0.06 | -0.38 | **0.69** |
| Cd | 0.37 | 0.11 | -0.04 | 1.00 | **0.72** | **0.69** | 0.47 | 0.18 | 0.15 | -0.13 | -0.34 |
| Pb | 0.33 | 0.18 | -0.07 | **0.72** | 1.00 | **0.95** | **0.65** | 0.24 | 0.34 | -0.27 | -0.35 |
| Zn | 0.50 | 0.42 | 0.07 | **0.69** | **0.95** | 1.00 | **0.82** | 0.40 | 0.47 | -0.45 | -0.17 |
| Cu | 0.62 | **0.76** | 0.32 | 0.47 | **0.65** | **0.82** | 1.00 | 0.51 | **0.68** | **-0.69** | 0.28 |
| Clay | **0.86** | **0.73** | 0.58 | 0.18 | 0.24 | 0.40 | 0.51 | 1.00 | 0.59 | **-0.89** | 0.17 |
| Silt | 0.33 | **0.78** | -0.06 | 0.15 | 0.34 | 0.47 | **0.68** | 0.59 | 1.00 | **-0.87** | 0.02 |
| Sand | **-0.69** | **-0.86** | -0.38 | -0.13 | -0.27 | -0.45 | **-0.69** | **-0.89** | **-0.87** | 1.00 | -0.24 |
| Gravel | 0.30 | 0.28 | **0.69** | -0.34 | -0.35 | -0.17 | 0.28 | 0.17 | 0.01 | -0.24 | 1.00 |

*Notes: Bolded values of correlation coefficient (r) are significant at the level of p < 0.05; negligible correlation for r = 0.00-0.09; weak correlation for r = 0.10-0.39; moderate correlation for r = 0.40-0.69; strong correlation for r = 0.70-0.89; very strong correlation for r = 0.90-1.00.*

Table S8. Correlation matrix between soil parameters at the Zdounky landfill

| r | pH | EC | Ni | Zn | Cu | Clay | Silt | Sand | Gravel |
| --- | --- | --- | --- | --- | --- | --- | --- | --- | --- |
| pH | 1.00 | **-0.93** | -0.76 | -0.46 | -0.47 | -0.20 | -0.49 | 0.17 | 0.69 |
| EC | **-0.93** | 1.00 | 0.61 | 0.29 | 0.45 | -0.14 | 0.37 | 0.11 | -0.57 |
| Ni | -0.76 | 0.61 | 1.00 | **0.87** | 0.77 | 0.53 | 0.58 | -0.48 | -0.73 |
| Zn | -0.46 | 0.29 | **0.87** | 1.00 | 0.78 | 0.48 | 0.22 | -0.33 | -0.35 |
| Cu | -0.47 | 0.44 | 0.77 | 0.78 | 1.00 | 0.22 | 0.12 | -0.11 | -0.26 |
| Clay | -0.20 | -0.14 | 0.53 | 0.48 | 0.22 | 1.00 | 0.61 | **-0.95** | -0.57 |
| Silt | -0.49 | 0.37 | 0.58 | 0.22 | 0.12 | 0.61 | 1.00 | -0.79 | **-0.97** |
| Sand | 0.17 | 0.11 | -0.48 | -0.33 | -0.11 | **-0.95** | -0.79 | 1.00 | 0.71 |
| Gravel | 0.69 | -0.57 | -0.73 | -0.35 | -0.26 | -0.57 | **-0.97** | 0.71 | 1.00 |

*Notes: Bolded values of correlation coefficient (r) are significant at the level of p < 0.05; negligible correlation for r = 0.00-0.09; weak correlation for r = 0.10-0.39; moderate correlation for r = 0.40-0.69; strong correlation for r = 0.70-0.89; very strong correlation for r = 0.90-1.00.*

Table S9. Carcinogenic and non-carcinogenic risks due to heavy metals exposure through direct soil ingestion (ADDing-soil).

| Point | Heavy metals | | | | |
| --- | --- | --- | --- | --- | --- |
|  | Ni | Cd | Pb | Zn | Cu |
|  | Carcinogenic risk | | | Non-carcinogenic risk | |
| *Radiowo* | | | | | |
| P-2A | 3.93E-06 | 8.98E-07 | 3.53E-06 | 3.06E-05 | 1.12E-05 |
| P-4 | 5.87E-08 | 3.87E-07 | 4.93E-06 | 2.24E-05 | 4.01E-06 |
| P-6 | 1.83E-06 | 8.34E-07 | 1.26E-05 | 6.38E-05 | 9.73E-06 |
| P-7 | 3.58E-06 | 1.29E-06 | 9.75E-06 | 5.74E-05 | 1.63E-05 |
| P-9 | 4.59E-06 | 1.02E-06 | 8.16E-06 | 7.13E-05 | 2.63E-05 |
| P-10 | 2.79E-06 | 7.28E-06 | 6.15E-05 | 1.74E-04 | 2.97E-05 |
| P-11A | 1.81E-06 | 6.28E-06 | 1.47E-05 | 7.29E-05 | 1.78E-05 |
| P-12 | 3.56E-06 | 9.75E-07 | 6.96E-06 | 6.04E-05 | 1.12E-05 |
| P-15 | 2.59E-06 | 8.16E-07 | 1.98E-05 | 6.31E-05 | 1.14E-05 |
| P-17 | 1.67E-06 | 7.05E-07 | 2.42E-05 | 1.06E-04 | 2.88E-05 |
| *Zdounky* | | | | | |
| MV-1 | 1.47E-06 | 5.87E-08 | 5.87E-08 | 4.10E-05 | 1.61E-05 |
| MV-2 | 3.93E-06 | 5.87E-08 | 5.87E-08 | 5.48E-05 | 3.55E-05 |
| MV-4 | 4.17E-06 | 5.87E-08 | 5.87E-08 | 6.38E-05 | 2.30E-05 |
| MV-5 | 3.87E-06 | 5.87E-08 | 5.87E-08 | 6.67E-05 | 2.34E-05 |
| MV-6 | 1.41E-06 | 5.87E-08 | 5.87E-08 | 7.12E-06 | 3.70E-06 |
| S-1 | 1.82E-06 | 5.87E-08 | 5.87E-08 | 3.00E-05 | 1.94E-05 |

*Notes: ADDing-soil values were calculated using IngR= 100 mg/day; EF = 350 days/year; ED = 30 years; CF = 0.000001 kg/mg; BW = 70 kg; AT_non-cancer_ = 10950; AT_cancer_ = 25550.*

Table S10. Carcinogenic and non-carcinogenic risk due to heavy metals exposure through inhalation of dust particles through mouth and nose (ADDinh).

| Point | Heavy metals | | | | |
| --- | --- | --- | --- | --- | --- |
|  | Ni | Cd | Pb | Zn | Cu |
|  | Carcinogenic risk | | | Non-carcinogenic risk | |
| *Radiowo* | | | | | |
| P-2A | 5.78E-10 | 1.32E-10 | 5.20E-10 | 4.50E-09 | 1.65E-09 |
| P-4 | 8.63E-12 | 5.70E-11 | 7.25E-10 | 3.29E-09 | 5.90E-10 |
| P-6 | 2.69E-10 | 1.23E-10 | 1.85E-09 | 9.38E-09 | 1.43E-09 |
| P-7 | 5.27E-10 | 1.90E-10 | 1.43E-09 | 8.44E-09 | 2.39E-09 |
| P-9 | 6.74E-10 | 1.49E-10 | 1.20E-09 | 1.05E-08 | 3.86E-09 |
| P-10 | 4.10E-10 | 1.07E-09 | 9.05E-09 | 2.57E-08 | 4.37E-09 |
| P-11A | 2.66E-10 | 9.24E-10 | 2.17E-09 | 1.07E-08 | 2.61E-09 |
| P-12 | 5.23E-10 | 1.43E-10 | 1.02E-09 | 8.89E-09 | 1.65E-09 |
| P-15 | 3.82E-10 | 1.20E-10 | 2.92E-09 | 9.28E-09 | 1.67E-09 |
| P-17 | 2.46E-10 | 1.04E-10 | 3.56E-09 | 1.56E-08 | 4.23E-09 |
| *Zdounky* | | | | | |
| MV-1 | 2.16E-10 | 8.63E-12 | 8.63E-12 | 6.02E-09 | 2.36E-09 |
| MV-2 | 5.78E-10 | 8.63E-12 | 8.63E-12 | 8.06E-09 | 5.22E-09 |
| MV-4 | 6.13E-10 | 8.63E-12 | 8.63E-12 | 9.39E-09 | 3.38E-09 |
| MV-5 | 5.70E-10 | 8.63E-12 | 8.63E-12 | 9.81E-09 | 3.44E-09 |
| MV-6 | 2.07E-10 | 8.63E-12 | 8.63E-12 | 1.05E-09 | 5.44E-10 |
| S-1 | 2.67E-10 | 8.63E-12 | 8.63E-12 | 4.41E-09 | 2.86E-09 |

*Notes: ADDinh values were calculated using InhR= 20 m^3^/day; EF = 350 days/year; ED = 30 years; BW = 70 kg; AT_non-cancer_ = 10950; AT_cancer_ = 25550, PEF = 1360000000 m^3^/kg.*

Table S11. Carcinogenic and non-carcinogenic due to heavy metals exposure through dermal absorption (ADDder calculated for Radiowo landfill).

| Point | Heavy metals | | | | |
| --- | --- | --- | --- | --- | --- |
|  | Ni | Cd | Pb | Zn | Cu |
|  | Carcinogenic risk | | | Non- carcinogenic risk | |
| *Radiowo* | | | | | |
| P-2A | 1.57E-08 | 3.58E-09 | 1.41E-08 | 1.22E-07 | 4.46E-08 |
| P-4 | 2.34E-10 | 1.55E-09 | 1.97E-08 | 8.93E-08 | 1.60E-08 |
| P-6 | 7.29E-09 | 3.33E-09 | 5.01E-08 | 2.54E-07 | 3.88E-08 |
| P-7 | 1.43E-08 | 5.15E-09 | 3.89E-08 | 2.29E-07 | 6.50E-08 |
| P-9 | 1.83E-08 | 4.05E-09 | 3.26E-08 | 2.84E-07 | 1.05E-07 |
| P-10 | 1.11E-08 | 2.90E-08 | 2.46E-07 | 6.97E-07 | 1.19E-07 |
| P-11A | 7.21E-09 | 2.51E-08 | 5.88E-08 | 2.91E-07 | 7.09E-08 |
| P-12 | 1.42E-08 | 3.89E-09 | 2.78E-08 | 2.41E-07 | 4.47E-08 |
| P-15 | 1.04E-08 | 3.26E-09 | 7.92E-08 | 2.52E-07 | 4.53E-08 |
| P-17 | 6.68E-09 | 2.81E-09 | 9.66E-08 | 4.23E-07 | 1.15E-07 |
| *Zdounky* | | | | | |
| MV-1 | 5.86E-09 | 2.34E-10 | 2.34E-10 | 1.63E-07 | 6.39E-08 |
| MV-2 | 1.57E-08 | 2.34E-10 | 2.34E-10 | 2.19E-07 | 1.42E-07 |
| MV-4 | 1.66E-08 | 2.34E-10 | 2.34E-10 | 2.54E-07 | 9.18E-08 |
| MV-5 | 1.55E-08 | 2.34E-10 | 2.34E-10 | 2.66E-07 | 9.35E-08 |
| MV-6 | 5.62E-09 | 2.34E-10 | 2.34E-10 | 2.84E-08 | 1.48E-08 |
| S-1 | 7.26E-09 | 2.34E-10 | 2.34E-10 | 1.20E-07 | 7.76E-08 |

*Notes: ADDder values were calculated using AFsoil = 0.07 mg/cm2/day; SA = 5700 cm2; ABS = 0.001; EF = 350 days/year; ED = 30 years; CF = 0.000001 kg/mg; BW = 70 kg; AT non-cancer = 10950; ATcancer = 25550, PEF = 1360000000 m^3^/kg.*

Table S12. HQ values calculated for different routes of exposure – Radiowo landfill.

| Point | Heavy metals | | | | |
| --- | --- | --- | --- | --- | --- |
|  | Ni | Cd | Pb | Zn | Cu |
| *Ingestion* | | | | | |
| P-2A | 4.58E-04 | 2.10E-03 | 2.36E-03 | 1.02E-04 | 2.80E-04 |
| P-4 | 6.85E-06 | 9.04E-04 | 3.28E-03 | 7.46E-05 | 1.00E-04 |
| P-6 | 2.13E-04 | 1.95E-03 | 8.36E-03 | 2.12E-04 | 2.43E-04 |
| P-7 | 4.18E-04 | 3.01E-03 | 6.50E-03 | 1.91E-04 | 4.07E-04 |
| P-9 | 5.35E-04 | 2.37E-03 | 5.44E-03 | 2.38E-04 | 6.57E-04 |
| P-10 | 3.25E-04 | 1.70E-02 | 4.10E-02 | 5.82E-04 | 7.42E-04 |
| P-11A | 2.11E-04 | 1.47E-02 | 9.80E-03 | 2.42E-04 | 4.44E-04 |
| P-12 | 4.15E-04 | 2.27E-03 | 4.64E-03 | 2.01E-04 | 2.80E-04 |
| P-15 | 3.03E-04 | 1.90E-03 | 1.32E-02 | 2.10E-04 | 2.84E-04 |
| P-17 | 1.95E-04 | 1.64E-03 | 1.61E-02 | 3.53E-04 | 7.19E-04 |
| *Inhalation* | | | | | |
| P-2A | 6.74E-08 | 3.08E-07 | 3.46E-07 | 1.50E-08 | 4.11E-08 |
| P-4 | 1.01E-09 | 1.33E-07 | 4.83E-07 | 1.10E-08 | 1.47E-08 |
| P-6 | 3.13E-08 | 2.86E-07 | 1.23E-06 | 3.12E-08 | 3.57E-08 |
| P-7 | 6.14E-08 | 4.43E-07 | 9.55E-07 | 2.81E-08 | 5.99E-08 |
| P-9 | 7.87E-08 | 3.48E-07 | 8.00E-07 | 3.49E-08 | 9.65E-08 |
| P-10 | 4.78E-08 | 2.50E-06 | 6.03E-06 | 8.55E-08 | 1.09E-07 |
| P-11A | 3.10E-08 | 2.15E-06 | 1.44E-06 | 3.57E-08 | 6.53E-08 |
| P-12 | 6.10E-08 | 3.34E-07 | 6.82E-07 | 2.96E-08 | 4.11E-08 |
| P-15 | 4.45E-08 | 2.80E-07 | 1.94E-06 | 3.09E-08 | 4.17E-08 |
| P-17 | 2.87E-08 | 2.41E-07 | 2.37E-06 | 5.20E-08 | 1.06E-07 |
| *Dermal absorption* | | | | | |
| P-2A | 4.57E-05 | 8.36E-04 | 6.21E-05 | 4.07E-07 | 1.12E-06 |
| P-4 | 6.83E-07 | 3.61E-04 | 8.66E-05 | 2.97E-07 | 4.00E-07 |
| P-6 | 2.12E-05 | 7.76E-04 | 2.20E-04 | 8.48E-07 | 9.70E-07 |
| P-7 | 4.16E-05 | 1.20E-03 | 1.71E-04 | 7.63E-07 | 1.63E-06 |
| P-9 | 5.33E-05 | 9.46E-04 | 1.43E-04 | 9.48E-07 | 2.62E-06 |
| P-10 | 3.24E-05 | 6.78E-03 | 1.08E-03 | 2.32E-06 | 2.96E-06 |
| P-11A | 2.10E-05 | 5.85E-03 | 2.59E-04 | 9.69E-07 | 1.77E-06 |
| P-12 | 4.14E-05 | 9.07E-04 | 1.22E-04 | 8.04E-07 | 1.12E-06 |
| P-15 | 3.02E-05 | 7.59E-04 | 3.49E-04 | 8.39E-07 | 1.13E-06 |
| P-17 | 1.95E-05 | 6.56E-04 | 4.26E-04 | 1.41E-06 | 2.87E-06 |

*Notes: HQ values were calculated using RfD_ing-soil_= RfD_inh_: 0.02 mg/kg/day (Ni); 0.001 mg/kg/day (Cd); 0.0035 mg/kg/day (Pb); 0.3 mg/kg/day (Zn);0.04 mg/kg/day (Cu); RfDder: 0.0008 mg/kg/day (Ni); 0.00001 mg/kg/day (Cd); 0.00053 mg/kg/day (Pb); 0.3 mg/kg/day (Zn); 0.04 mg/kg/day (Cu).*

Table S13. HQ values calculated for different routes of exposure – Zdounky landfill.

| Point | Heavy metals | | | | |
| --- | --- | --- | --- | --- | --- |
|  | Ni | Cd | Pb | Zn | Cu |
| *Ingestion* | | | | | |
| MV-1 | 1.71E-04 | 1.37E-04 | 3.92E-05 | 1.36E-04 | 4.01E-04 |
| MV-2 | 4.59E-04 | 1.37E-04 | 3.92E-05 | 1.83E-04 | 8.87E-04 |
| MV-4 | 4.86E-04 | 1.37E-04 | 3.92E-05 | 2.13E-04 | 5.75E-04 |
| MV-5 | 4.52E-04 | 1.37E-04 | 3.92E-05 | 2.22E-04 | 5.86E-04 |
| MV-6 | 1.64E-04 | 1.37E-04 | 3.92E-05 | 2.37E-05 | 9.25E-05 |
| S-1 | 2.12E-04 | 1.37E-04 | 3.92E-05 | 1.00E-04 | 4.86E-04 |
| *Inhalation* | | | | | |
| MV-1 | 2.52E-08 | 2.01E-08 | 5.75E-09 | 2.01E-08 | 5.89E-08 |
| MV-2 | 6.75E-08 | 2.01E-08 | 5.75E-09 | 2.69E-08 | 1.30E-07 |
| MV-4 | 7.15E-08 | 2.01E-08 | 5.75E-09 | 3.13E-08 | 8.46E-08 |
| MV-5 | 6.65E-08 | 2.01E-08 | 5.75E-09 | 3.27E-08 | 8.61E-08 |
| MV-6 | 2.42E-08 | 2.01E-08 | 5.75E-09 | 3.49E-09 | 1.36E-08 |
| S-1 | 3.12E-08 | 2.01E-08 | 5.75E-09 | 1.47E-08 | 7.15E-08 |
| *Dermal absorption* | | | | | |
| MV-1 | 1.71E-05 | 5.46E-05 | 1.03E-06 | 5.44E-07 | 1.60E-06 |
| MV-2 | 4.58E-05 | 5.46E-05 | 1.03E-06 | 7.29E-07 | 3.54E-06 |
| MV-4 | 4.85E-05 | 5.46E-05 | 1.03E-06 | 8.49E-07 | 2.30E-06 |
| MV-5 | 4.51E-05 | 5.46E-05 | 1.03E-06 | 8.87E-07 | 2.34E-06 |
| MV-6 | 1.64E-05 | 5.46E-05 | 1.03E-06 | 9.47E-08 | 3.69E-07 |
| S-1 | 2.12E-05 | 5.46E-05 | 1.03E-06 | 3.99E-07 | 1.94E-06 |

*Notes: HQ values were calculated using RfD_ing-soil_= RfD_inh_: 0.02 mg/kg/day (Ni); 0.001 mg/kg/day (Cd); 0.0035 mg/kg/day (Pb); 0.3 mg/kg/day (Zn);0.04 mg/kg/day (Cu); RfDder: 0.0008 mg/kg/day (Ni); 0.00001 mg/kg/day (Cd); 0.00053 mg/kg/day (Pb); 0.3 mg/kg/day (Zn); 0.04 mg/kg/day (Cu).*

Table S14. CR values for the Radiowo landfill.

| Point | Heavy metals | | |
| --- | --- | --- | --- |
|  | Ni | Cd | Pb |
| *Ingestion* | | | |
| P-2A | 6.68E-06 | 3.41E-07 | 3.00E-08 |
| P-4 | 9.98E-08 | 1.47E-07 | 4.19E-08 |
| P-6 | 3.10E-06 | 3.17E-07 | 1.07E-07 |
| P-7 | 6.09E-06 | 4.91E-07 | 8.28E-08 |
| P-9 | 7.79E-06 | 3.86E-07 | 6.94E-08 |
| P-10 | 4.74E-06 | 2.77E-06 | 5.23E-07 |
| P-11A | 3.07E-06 | 2.39E-06 | 1.25E-07 |
| P-12 | 6.05E-06 | 3.70E-07 | 5.92E-08 |
| P-15 | 4.41E-06 | 3.10E-07 | 1.69E-07 |
| P-17 | 2.84E-06 | 2.68E-07 | 2.06E-07 |
| *Inhalation* | | | |
| P-2A | 5.20E-10 | 8.32E-10 | 2.18E-11 |
| P-4 | 7.77E-12 | 3.59E-10 | 3.05E-11 |
| P-6 | 2.42E-10 | 7.72E-10 | 7.75E-11 |
| P-7 | 4.74E-10 | 1.20E-09 | 6.02E-11 |
| P-9 | 6.07E-10 | 9.41E-10 | 5.04E-11 |
| P-10 | 3.69E-10 | 6.74E-09 | 3.80E-10 |
| P-11A | 2.39E-10 | 5.82E-09 | 9.11E-11 |
| P-12 | 4.71E-10 | 9.03E-10 | 4.30E-11 |
| P-15 | 3.43E-10 | 7.56E-10 | 1.23E-10 |
| P-17 | 2.21E-10 | 6.53E-10 | 1.49E-10 |
| *Dermal absorption* | | | |
| P-2A | 6.66E-07 | 1.36E-09 | 1.20E-10 |
| P-4 | 9.96E-09 | 5.87E-10 | 1.67E-10 |
| P-6 | 3.10E-07 | 1.26E-09 | 4.26E-10 |
| P-7 | 6.07E-07 | 1.96E-09 | 3.31E-10 |
| P-9 | 7.78E-07 | 1.54E-09 | 2.77E-10 |
| P-10 | 4.73E-07 | 1.10E-08 | 2.09E-09 |
| P-11A | 3.07E-07 | 9.52E-09 | 5.00E-10 |
| P-12 | 6.03E-07 | 1.48E-09 | 2.36E-10 |
| P-15 | 4.40E-07 | 1.24E-09 | 6.73E-10 |
| P-17 | 2.84E-07 | 1.07E-09 | 8.21E-10 |

Table S15. CR values for the Zdounky landfill.

| Point | Heavy metals | | |
| --- | --- | --- | --- |
|  | Ni | Cd | Pb |
| Ingestion | | | |
| MV-1 | 2.50E-06 | 2.23E-08 | 4.99E-10 |
| MV-2 | 6.69E-06 | 2.23E-08 | 4.99E-10 |
| MV-4 | 7.09E-06 | 2.23E-08 | 4.99E-10 |
| MV-5 | 6.59E-06 | 2.23E-08 | 4.99E-10 |
| MV-6 | 2.39E-06 | 2.23E-08 | 4.99E-10 |
| S-1 | 3.09E-06 | 2.23E-08 | 4.99E-10 |
| Inhalation | | | |
| MV-1 | 1.94E-10 | 5.44E-11 | 3.63E-13 |
| MV-2 | 5.20E-10 | 5.44E-11 | 3.63E-13 |
| MV-4 | 5.52E-10 | 5.44E-11 | 3.63E-13 |
| MV-5 | 5.13E-10 | 5.44E-11 | 3.63E-13 |
| MV-6 | 1.86E-10 | 5.44E-11 | 3.63E-13 |
| S-1 | 2.41E-10 | 5.44E-11 | 3.63E-13 |
| Dermal absorption | | | |
| MV-1 | 2.49E-07 | 8.90E-11 | 1.99E-12 |
| MV-2 | 6.67E-07 | 8.90E-11 | 1.99E-12 |
| MV-4 | 7.07E-07 | 8.90E-11 | 1.99E-12 |
| MV-5 | 6.57E-07 | 8.90E-11 | 1.99E-12 |
| MV-6 | 2.38E-07 | 8.90E-11 | 1.99E-12 |
| S-1 | 3.09E-07 | 8.90E-11 | 1.99E-12 |
